# Structure-based prediction of nucleic acid binding residues by merging deep learning- and template-based approaches

**DOI:** 10.1371/journal.pcbi.1011428

**Published:** 2023-09-06

**Authors:** Zheng Jiang, Yue-Yue Shen, Rong Liu

**Affiliations:** Hubei Key Laboratory of Agricultural Bioinformatics, College of Informatics, Huazhong Agricultural University, Wuhan, China; Sun Yat-Sen University, CHINA

## Abstract

Accurate prediction of nucleic binding residues is essential for the understanding of transcription and translation processes. Integration of feature- and template-based strategies could improve the prediction of these key residues in proteins. Nevertheless, traditional hybrid algorithms have been surpassed by recently developed deep learning-based methods, and the possibility of integrating deep learning- and template-based approaches to improve performance remains to be explored. To address these issues, we developed a novel structure-based integrative algorithm called NABind that can accurately predict DNA- and RNA-binding residues. A deep learning module was built based on the diversified sequence and structural descriptors and edge aggregated graph attention networks, while a template module was constructed by transforming the alignments between the query and its multiple templates into features for supervised learning. Furthermore, the stacking strategy was adopted to integrate the above two modules for improving prediction performance. Finally, a post-processing module dependent on the random walk algorithm was proposed to further correct the integrative predictions. Extensive evaluations indicated that our approach could not only achieve excellent performance on both native and predicted structures but also outperformed existing hybrid algorithms and recent deep learning methods. The NABind server is available at http://liulab.hzau.edu.cn/NABind/.

## Author summary

Ten years ago we developed two hybrid algorithms (DNABind and RBRDetector) to predict nucleic acid binding residues by combining machine learning- and template-based strategies. However, this kind of algorithms have been surpassed by recent deep learning methods. Moreover, the interplay between deep learning- and template-based approaches has yet to be explored. We thus designed a new generation hybrid algorithm termed NABind, in which a deep learning module was established by using diversified sequence and structural descriptors and edge-featured graph attention networks, while a template module was created by exploiting the relationship between the query protein and its multiple templates for supervised learning. Afterward, a merging module based on the stacking strategy was adopted to integrate the above two modules, and a post-processing module dependent on the random walk algorithm was utilized to correct the integrative predictions. The new algorithm outperformed traditional hybrid methods by a large margin and showed better results than purely deep learning-based methods.

## Introduction

Protein-nucleic acid interactions are fundamental to transcription and translation processes [1,2]. For example, transcription factors that are specific DNA-binding proteins (DBPs) could regulate gene expression by binding DNA fragments such as promoters and enhancers [3]. A great number of translational regulators are RNA-binding proteins (RBPs), which regulate translation initiation using the location of their binding sites on the mRNA [4]. Identification of nucleic acid binding sites in proteins could be helpful in understanding the mechanisms underlying these key biological processes. Different experimental techniques, such as X-ray crystallography, nuclear magnetic resonance and electron microscopy, have been used to determine the binding modes between proteins and nucleic acids, but these methods are costly and time-consuming. Thus, there is a pressing need to develop computational algorithms for predicting DNA- and RNA-binding residues (DBRs and RBRs).

Over the past two decades, a series of algorithms have been developed in this field and could be classified into three categories based on the underlying strategies, namely feature-based algorithms, template-based algorithms and hybrid algorithms [5–9]. Feature-based algorithms could infer binding residues through customized scoring functions, machine learning methods, or deep learning methods. Corsi *et al*. designed a scoring function that comprised the circular variance features, sequence conservation, and physicochemical properties to identify DBRs [10]. Developers of DNAPred, RNABindR, and SVMnuc applied different machine learning algorithms to nucleic acid binding residue prediction [11–13]. Xia *et al*. integrated hierarchical graph neural networks with sequence and structural features (GraphBind) to predict DBRs and RBRs [14]. Yuan *et al*. proposed GraphSite which used representations generated by AlphaFold2 and graph transformers to yield a relatively higher accuracy for DBRs [15]. Li *et al*. combined protein surface topography with deep residue networks (PSTPRNA) to predict RBRs [16]. The primary advantage of feature-based algorithms is that they could provide effective predictions for most query proteins.

Template-based algorithms identify nucleic acid binding residues by mapping binding information from template proteins to query proteins. Based on sequence or structural information, this kind of methods can use different techniques for searching templates. For instance, Yang *et al*. developed S-SITE that adopted sequence profile-profile alignments to detect templates [17]. The structure-based methods, such as TM-SITE and COFACTOR, used TMalign as the search engine for retrieving the templates of DBPs and RBPs [17,18]. When the reliable templates are available for query proteins, template-based algorithms could achieve highly accurate prediction results. However, these approaches might become less effective if the suitable templates are unavailable. To utilize the complementarity between feature- and template-based methods, several hybrid algorithms have been developed in this field. For instance, ten years ago we developed two algorithms, namely DNABind and RBRDetector, which adopted a piecewise function in conjunction with the weighted combination to merge machine learning- and template-based strategies for DBR and RBR predictions, respectively [19,20]. Su *et al*. proposed NucBind which selected the output of feature- or template-based module as the final result according to the template quality [13]. Although existing hybrid algorithms resulted in better performance compared with the previous state-of-the-art counterparts, this kind of methods have been surpassed by recently developed deep learning-based algorithms, such as GraphBind and GraphSite [14,15]. Furthermore, the possibility of merging deep learning and template approaches to improve performance has yet to be explored. Collectively, it is highly desirable to develop a new generation hybrid algorithm to address the above limitations.

In this work, we designed a novel structure-based algorithm termed NABind to predict DBRs and RBRs. In this algorithm, a deep learning module was created by using the diversified sequence and structural features and edge aggregated graph attention networks (EGAT), while a template module was constructed by transforming the relationship between the query and its multiple templates for supervised learning. To further improve prediction performance, a merging module based on the stacking strategy was adopted to combine the above two modules, and a post-processing module dependent on the random walk algorithm was used to correct the integrative results. Evaluated on both native and predicted structures, NABind not only achieved better performance than traditional hybrid algorithms by a large margin but also outperformed purely deep learning-based methods.

## Results

### Overview of the NABind algorithm

**Fig 1:**
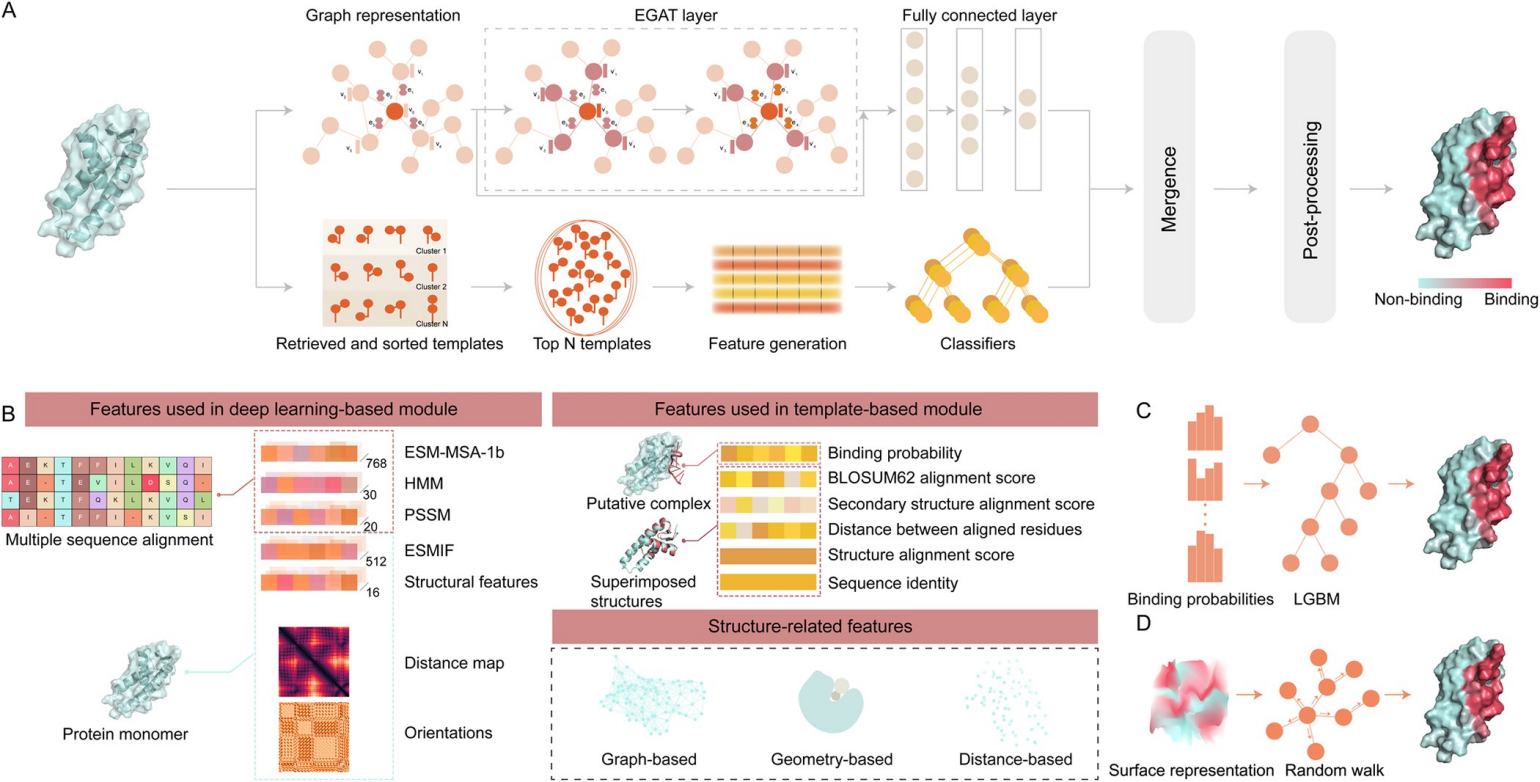
Flowchart of NABind algorithm. (A) NABind comprising four basic modules (i.e. a deep learning-based module, a template-based module, a merging module and a post-processing module). In the deep learning module, proteins were represented as graphs which were then fed into the EGAT layer and fully connected layer for node classification. In the template module, multiple templates were retrieved for each query, and template-related features were generated for supervised learning. (B) Features used in different modules. For deep learning, node features included sequence descriptors and structural descriptors, while edge features included the distance and orientation between residues. The template features included overall alignment descriptors and residue-based alignment descriptors. (C) Schematics of merging module. This module was implemented by the LGBM method, the inputs of which were the outputs of deep learning and template modules. (D) Schematics of post-processing module. In this module, the random walk process was performed on the surface residue network to optimize binding probabilities.

As shown in Fig 1A, the proposed algorithm is composed of four parts: a deep learning-based module, a template-based module, a merging module, and a post-processing module. Within the deep learning phase, the protein structure was converted into a graph representation, the structural context of each residue was learned by EGAT, and the binding probabilities of residues were assigned by the fully connected layer. Especially, diversified sequence and structural descriptors, including embeddings from protein language models and other hand-crafted features, were extracted and assigned to the nodes and edges of the graph (Fig 1B). Regarding the template section, this module searched the query structure against the template library to obtain suitable templates, generated template-related features for each residue based on query-template pairs (Fig 1B), and predicted the binding probabilities of residues through machine learning classifiers. Afterwards, the stacking strategy was adopted to integrate the deep learning and template modules (Fig 1C). Using the output results of the above two modules as features, an ensemble classifier was established to generate the binding probabilities of residues in the merging phase. Finally, the post-processing module was utilized to correct the outputs of the merging module by performing random walks on networks comprising protein surface residues (Fig 1D).

### Coupling structural features with sequence features boosted predictions

**Fig 2:**
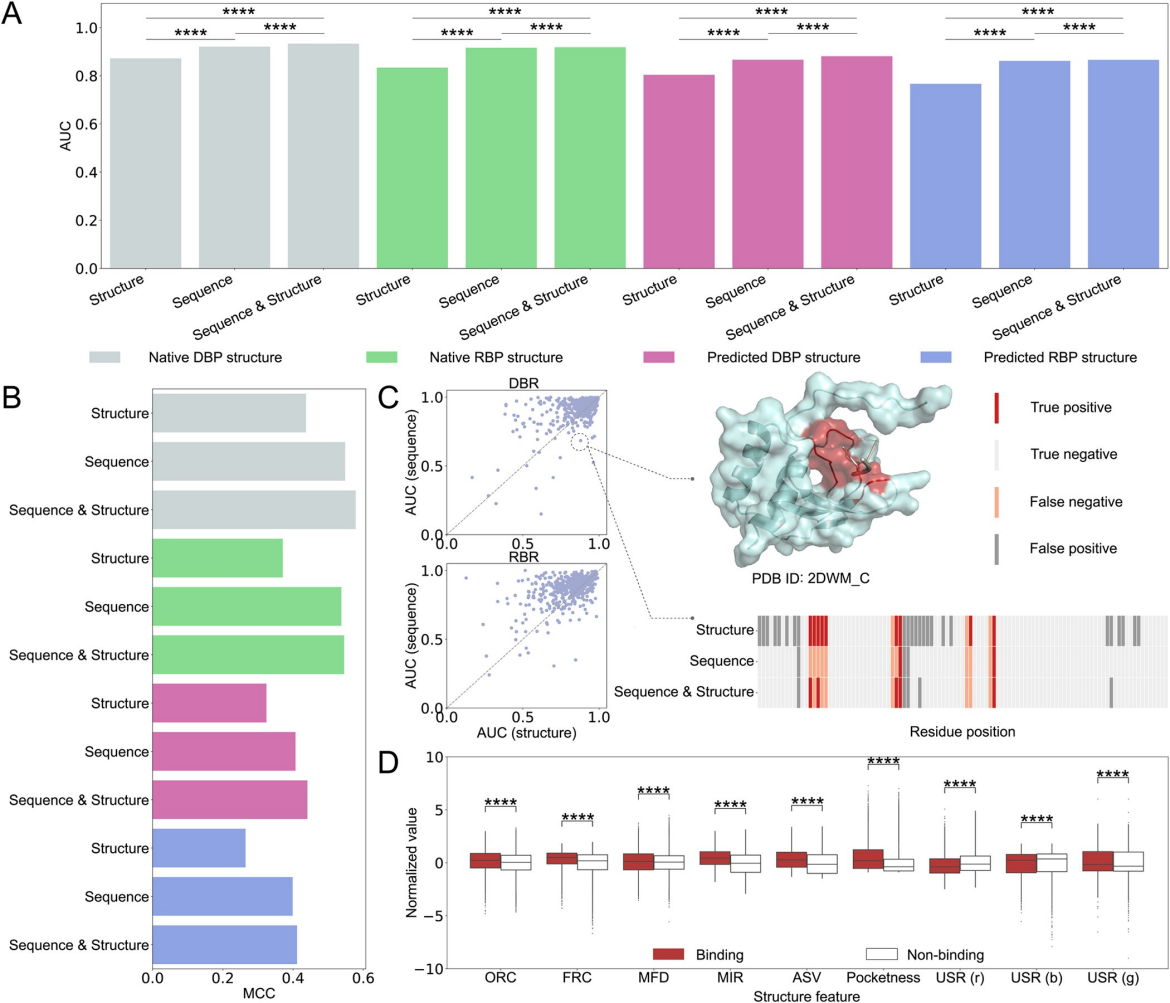
Comparison of deep learning models using different types of features and comparison of structural features of binding and non-binding residues. (A) AUC measures for different types of features. Significance tests were performed as described in the Methods section. (B) MCC measures for different types of features. (C) Scatter plots of AUC for native structures and an example with prediction results generated by different types of features. (D) Comparison of partial structural features between DNA-binding and non-binding residues. The complete comparison is presented in S2 Fig. ORC: Ollivier Ricci curvature, FRC: Forman Ricci curvature, MFD: multifractal dimension, MIR: minimum inaccessible radius, ASV: accessible shell volume, and USR: ultrafast shape recognition. Significant differences were evaluated using Wilcoxon rank sum test. **** *p* < 0.0001, *** 0.0001 ≤ *p* < 0.001, ** 0.001 ≤ *p* < 0.01, * 0.01 ≤ *p* < 0.05 and ns: *p* ≥ 0.05.

Based on the training sets (DBR_573 and RBR_495), we assessed the performance of deep learning module using 5-fold cross-validation. Unless noted otherwise, the performance represents the results of protein-based evaluations (see Methods). Because only limited structural information was used in previous deep learning-based methods (e.g. GraphBind and GraphSite [14,15]), a group of novel structural descriptors were explored in this work. Moreover, we compared the effectiveness of structural features and sequence features and investigated the complementarity between these two types of features. Fig 2A and 2B combined with S1 Fig and S1 Table shows the performances of different models on the training sets. By leveraging powerful MSA embeddings, sequence features achieved favorable results. Structural features yielded relatively worse performance than sequence features for both native and predicted structures. For instance, the differences in the MCC, AUC and AUPR measures were approximately 0.10, 0.05 and 0.10 for DBRs. Meanwhile, a systematic comparison of structural features between binding and non-binding residues was performed to check whether certain binding patterns could be captured. Compared to non-binding residues, binding residues tended to be surrounded by a denser context in terms of graph-based descriptors, be located in pocket regions in terms of geometry-based descriptors, and be closer to the center of proteins in terms of distance-based descriptors (Figs 2D and S2). Furthermore, compared to using sequence features alone, the combination of structural and sequence features could achieve better performance by approximately 5% in MCC, 2% in AUC, and 5% in AUPR for both DBPs and RBPs. S1B Fig shows that the density curve of the AUC and AUPR of chains was shifted toward higher values based on the merged features. The prediction results of a DBP (PDB ID: 2DWM_C) are shown in Fig 2C. In this example, structural features recovered more binding residues than sequence features, thus improving overall performance. S1C Fig shows the number of binding residues in the whole datasets recovered by the two types of features. In summary, although the utility of structural descriptors was relatively weaker, they could provide useful clues to binding residues that cannot be identified using sequence descriptors individually.

### Effectiveness of basic modules

**Fig 3:**
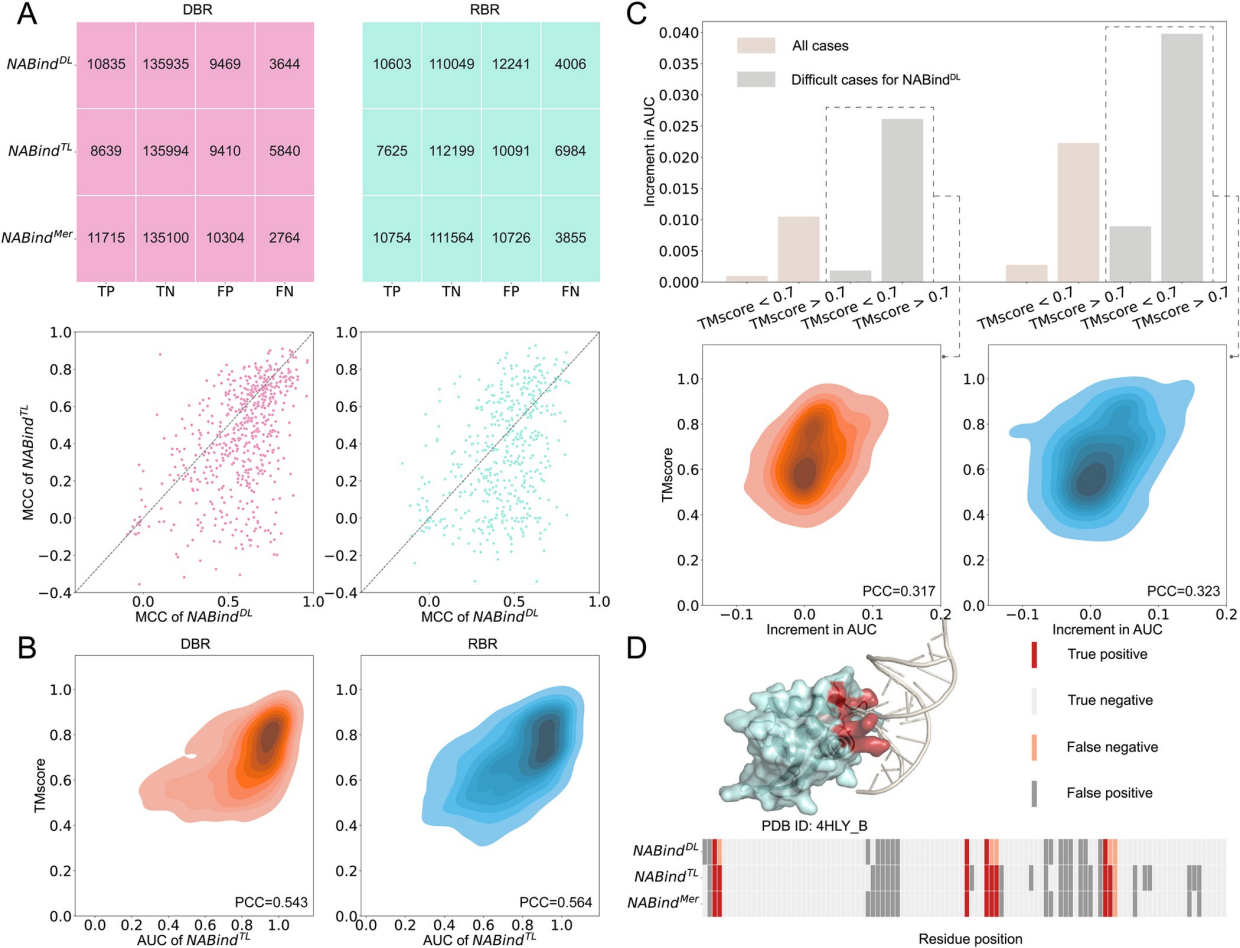
Usefulness of template-based module and merging module. (A) Performance comparison of the deep learning-based module, template-based module, and merging module. (B) Relationship between the AUC of chains and the TM-score of the best template. (C) Increments in AUC by incorporating template-based predictions and the relationship between the increment in AUC of difficult cases and the TM-score of the best template. Difficult cases denote those chains in the deep learning phase with AUC values less than the average AUC of all chains. (D) An example with prediction results of NABind^DL^, NABind^TL^ and NABind^Mer^.

In this section, we evaluated NABind on DBR_573 and RBR_495. The results of NABind and its basic modules are shown in Table 1. For the native protein structures, the deep learning module outperformed the template module by approximately 0.10 in the MCC, AUC and AUPR measures. Despite the overall advantage of the deep learning module, the binding sites in a significant fraction of proteins were better predicted by the template module, as shown in the scatter plots of Figs 3A and S3. By merging the two modules, the MCC, AUC and AUPR measures were increased from 0.575, 0.931 and 0.695 to 0.595, 0.939 and 0.728 on DBR_573 and from 0.441, 0.880 and 0.581 to 0.473, 0.893 and 0.621 on RBR_495. The P-values of statistical significance tests are shown in S2 Table. The merging module could obtain more true positives, especially an increment of 880 residues for DBR_573 (Fig 3A). To further illustrate the impact of template module on the overall performance, we showed that the predictive strength of this module was correlated with the quality of retrieved templates. As shown in Fig 3B, the Pearson correlation coefficients between the AUC of chains and the TMscore of the best template were 0.543 and 0.564 for DBR_573 and RBR_495, respectively. Then, the chains in each dataset were divided into two groups, namely those with good templates (TMscore > 0.7) and those without good templates (TMscore < 0.7), to observe the increment in AUC by incorporating the template module. This analysis was performed on the whole datasets as well as on the subsets where the deep learning module had poor performance. Here, if the AUC of a chain was lower than the average AUC of all chains, this chain was considered to be a difficult case for the deep learning method. Fig 3C displays that the merging module could clearly improve the AUC measure of chains having high quality templates. This tendency was especially remarkable for the difficult cases. Meanwhile, the correlation coefficients between the increment in AUC and the TMscore of the best template were 0.317 and 0.323 for DBPs and RBPs, respectively. These results indicated that the template module could serve as a complement to the deep learning module. Fig 3D shows an example to illustrate this point. Compared to the deep learning module that achieved only 4 true positives, the merging module recovered 8 binding residues by incorporating template information. Subsequently, the results of the merging model were further corrected by the random walk algorithm on protein surface residues. Due to the post-processing procedure, the evaluation measures were slightly improved on both DBR and RBR datasets (Table 1). As shown in S4 Fig, the major contribution of this process could be the reduction of false positives generated by the merging module, which would be useful in guiding biological experiments.

**Table 1:** Performance of different modules on training sets using 5-fold cross-validation.

Dataset	Classifier	Recall	Precision	F1	MCC	AUC	AUPR
DBR_573	NABind^DL^	0.748	0.549	0.606	0.575	0.931	0.695
	NABind^TL^	0.610	0.479	0.502	0.455	0.831	0.561
	NABind^Mer^	0.812	0.538	0.622	0.595	0.939	0.728
	NABind	0.754	0.587	0.634	0.606	0.940	0.729
DBR_573*	NABind^DL^	0.713	0.532	0.581	0.545	0.918	0.658
	NABind^TL^	0.490	0.405	0.404	0.349	0.774	0.464
	NABind^Mer^	0.759	0.510	0.582	0.549	0.920	0.666
	NABind	0.740	0.524	0.584	0.550	0.920	0.666
RBR_495	NABind^DL^	0.697	0.454	0.516	0.441	0.880	0.581
	NABind^TL^	0.539	0.427	0.420	0.347	0.768	0.481
	NABind^Mer^	0.723	0.482	0.541	0.473	0.893	0.621
	NABind	0.733	0.474	0.538	0.470	0.894	0.622
RBR_495*	NABind^DL^	0.677	0.430	0.492	0.410	0.864	0.548
	NABind^TL^	0.495	0.356	0.352	0.255	0.719	0.405
	NABind^Mer^	0.725	0.416	0.493	0.413	0.870	0.561
	NABind	0.662	0.459	0.500	0.424	0.871	0.563

* represents trRosetta-based predicted protein structures used for evaluation. DL denotes the deep learning-based module, TL denotes the template-based module, and Mer denotes the merging module.

Furthermore, we evaluated our method using predicted protein structures. The deep learning module was still superior to the template module on the predicted structures, but the differences in evaluation metrics were more remarkable. Regarding the template method, the results for predicted structures were clearly worse than those for native structures. For instance, the MCC, AUC and AUPR were decreased from 0.347, 0.768 and 0.481 to 0.255, 0.719 and 0.405 on RBR_495. S5 Fig displays that there was a negative correlation between the similarity of predicted and native structures and the change in the performance of template method on the two types of structures. In other words, as the structural similarity decreased, the performance difference increased remarkably. In contrast, the deep learning method exhibited promising reliability when applied to the predicted structures. This suggested that the template method was more sensitive to structural changes than the deep learning method. Additionally, the effectiveness of our merging module and post-processing module was further evidenced by the predicted structures. Finally, NABind achieved the MCC, AUC and AUPR of 0.550, 0.920 and 0.666 on DBR_573 and 0.424, 0.871 and 0.563 on RBR_495, respectively. Based on the native and predicted structures, the better results were observed on DBR_573 than RBR_495 for both NABind^DL^ and NABind^TL^, which could be attributed to the following reasons. First, compared with DNAs, RNAs possess more flexible and diversified structures, which could lead to less conserved binding modes for RBPs than DBPs. The accurate prediction of RBRs thus becomes more challenging. Second, the structures of DBPs were more abundant than those of RBPs in training sets. This may result in a more accurate deep learning module for DBRs. Third, the template library of protein-DNA complexes could be more complete than that of protein-RNA complexes. This resulted in a better template module for DBRs. Merging the two basic modules, NABind performed more favorably on DBRs than RBRs.

### Improvements over our previous hybrid methods

**Fig 4:**
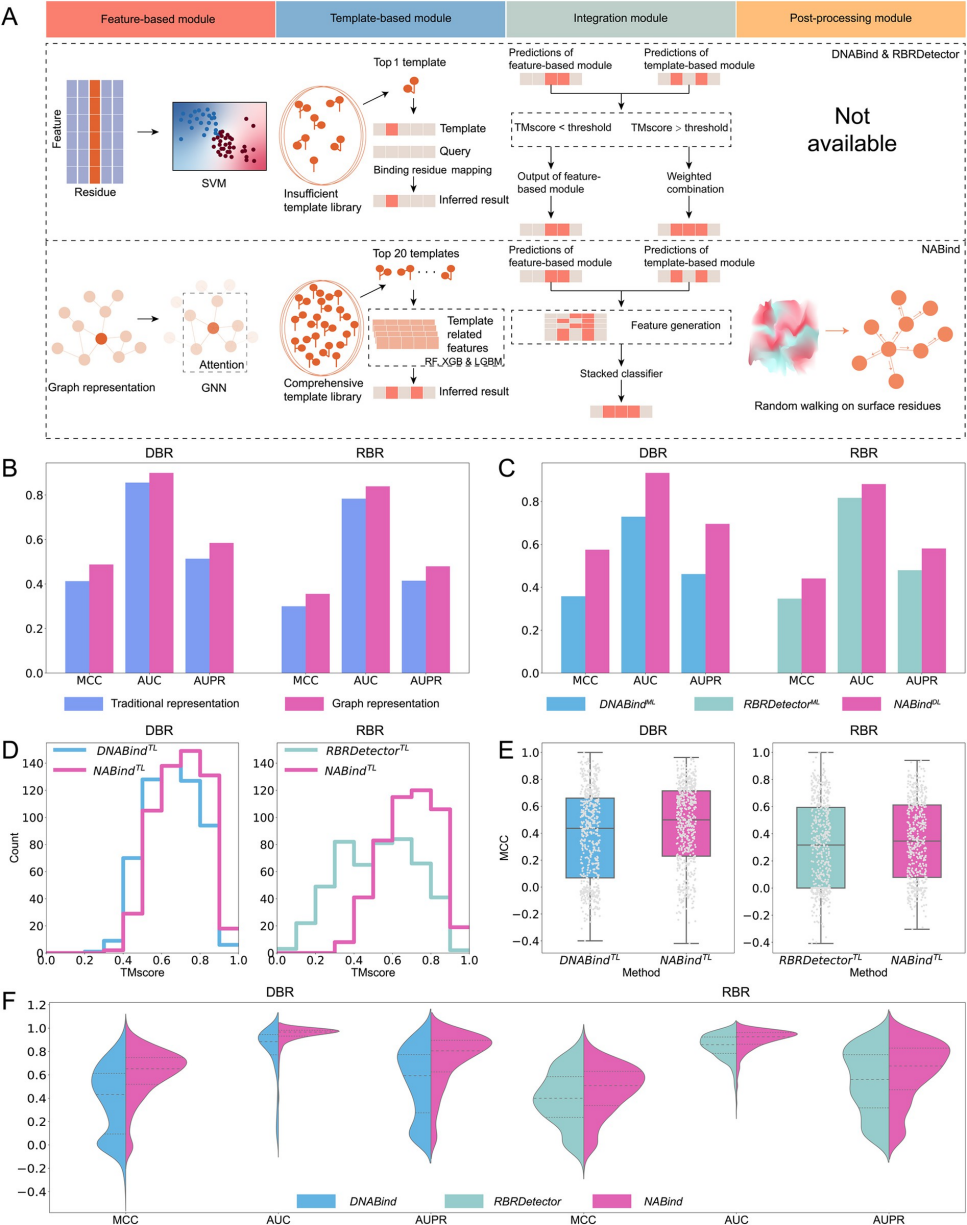
Comparison between NABind and our previous methods (i.e. DNABind and RBRDetector). (A) Differences in the design strategy of each module. The feature-based module adopted different feature representations and supervised learning models. The template-based module utilized different approaches for constructing the template library and inferring binding residues based on retrieved templates. The integration module used the stacking strategy instead of a piecewise function. A newly designed post-processing module was used in the updated method. (B) Comparison of improved and traditional residue representations using random forest classifiers. (C) Performance comparison of NABind^DL^, DNABind^ML^ and RBRDetector^ML^. (D) Statistics of best templates retrieved by current and previous methods. (E) Performance comparison of NABind^TL^, DNABind^TL^ and RBRDetector^TL^. (F) Performance comparison of NABind and our previous methods.

We adopted our previous algorithms, namely DNABind and RBRDetector [19,20], as baseline methods, which also explored the hybrid strategy to predict DBRs and RBRs, respectively. A head-to-head comparison of NABind and these two methods was performed on DBR_573 and RBR_495 to elucidate the improvements in this study. Fig 4A shows the detailed differences in designs for each module. For the feature-based module, the vast majority of features in NABind were different from those used in previous methods and were newly used in this field. Then, the prediction engine was the deep learning model (i.e. EGAT) instead of the machine learning model (i.e. SVM). Especially, the new model used the attention mechanism to learn the graph representation of each residue, whereas the traditional models indiscriminately adopted the features of neighboring residues. Combining the new features with the two types of representations, the former got better performance (Fig 4B). The comparison of these three feature-based modules showed that NABind^DL^ remarkably outperformed DNABind^ML^ and RBRDetector^ML^ (Fig 4C). For the template-based method, the new module did not use the training set as the template library but constructed a comprehensive independent library, which allowed NABind^TL^ to retrieve more high-quality templates (Fig 4D). Additionally, the current template-based prediction no longer simply relied on the predicted complex inspired by the best template, but took into account the alignment consistency between the query and its multiple templates, which were then converted into template-related features for supervised learning. Based on these updates, NABind^TL^ achieved more favorable results than DNABind^TL^ and RBRDetector^TL^ (Fig 4E and S3 Table). For the integration module, the current method used a stacking strategy rather than a piecewise function combined with the weighted combination used previously. As shown in S6C and S6D Fig, the use of stacking yielded slightly better results compared to the traditional method. Finally, NABind included a post-processing procedure which was neglected in the baseline methods. Fig 4F shows that NABind performed better than the baseline methods on native structures. The similar trends were also observed for predicted structures (S3 Table).

### NABind outperformed existing state-of-the-art methods

To further demonstrate the advantages of our method, we evaluated NABind and other state-of-the-art methods on the test sets (DBR_129, DBR_181, RBR_117 and RBR_106). As shown in Table 2 and S4 Table, NABind generated the best performance on all these four datasets by both protein- and residue-based evaluations, especially outperforming the traditional hybrid methods (e.g. NucBind, DNABind and RBRDetector) by a large margin. Then, we closely compared NABind with recently developed deep learning methods. For the native structures, all the measures of NABind were better than those of GraphBind (the third best DBR predictor and the second best RBR predictor) and GraphSite (the second best DBR predictor). In terms of the MCC and AUPR, especially, NABind surpassed these two methods by approximately 0.100. For the trRosetta-based predicted structures, the performance difference between NABind and GraphSite decreased, while the difference between NABind and GraphBind increased. For example, the AUCs of NABind, GraphSite, and GraphBind were 0.946, 0.920, and 0.914 for the native structures in DBR_129, while the measures were 0.925, 0.912, and 0.879 for the predicted structures. Regarding the AlphaFold2-based predicted structures, as shown in S5 and S6 Tables, our method still performed more favorably than the above two approaches and other deep learning algorithms (e.g. NCBRpred, NucleicNet and PSTPRNA). Furthermore, we compared NABind^DL^ with the top-ranked deep learning methods, and our models achieved generally better measures on the test sets (S7 Fig). Besides, the performance of each module in our algorithm is provided in S7 Table. The superior performance of NABind could be attributed to the following two factors: (a) we integrated multifaceted descriptors (e.g. embeddings from protein language models and novel structural features) and EGAT to build a powerful deep learning model and (b) we effectively incorporated the template-based prediction results and designed a post-processing module to improve performance.

**Table 2:** Comparison of NABind and existing methods on test sets.

Dataset	Method	Recall	Precision	F1	MCC	AUC	AUPR
DBR_129	COACH-D*	0.300	0.251	0.256	0.213	0.652	0.276
	COACH-D	0.339	0.291	0.291	0.253	0.679	0.313
	NCBRPred	0.590	0.268	0.331	0.300	0.817	0.360
	SVMnuc	0.595	0.282	0.352	0.320	0.821	0.364
	NucBind*	0.590	0.286	0.357	0.327	0.816	0.378
	NucBind	0.600	0.282	0.352	0.333	0.824	0.376
	DNABind*	0.689	0.322	0.405	0.383	0.864	0.456
	DNABind	0.683	0.383	0.455	0.438	0.885	0.476
	GraphBind*	0.614	0.377	0.420	0.403	0.879	0.484
	GraphBind	0.660	0.417	0.472	0.458	0.914	0.549
	GraphSite*	0.566	0.423	0.441	0.425	0.912	0.536
	GraphSite	0.584	0.471	0.466	0.455	0.920	0.560
	NABind*	**0.708**	0.420	0.494	0.482	0.925	0.560
	NABind	0.700	**0.504**	**0.541**	**0.535**	**0.946**	**0.637**
DBR_181	COACH-D*	0.254	0.199	0.203	0.171	0.637	0.222
	COACH-D	0.297	0.209	0.225	0.198	0.660	0.239
	NCBRPred	0.441	0.238	0.264	0.240	0.798	0.296
	SVMnuc	0.545	0.216	0.282	0.260	0.802	0.285
	NucBind*	0.544	0.222	0.286	0.265	0.802	0.288
	NucBind	0.540	0.227	0.290	0.269	0.802	0.290
	DNABind*	0.626	0.230	0.304	0.295	0.839	0.342
	DNABind	0.598	0.290	0.349	0.339	0.871	0.351
	GraphBind*	0.465	0.289	0.312	0.297	0.836	0.346
	GraphBind	0.566	0.331	0.379	0.370	0.895	0.421
	GraphSite*	0.454	0.343	0.345	0.332	0.892	0.413
	GraphSite	0.488	0.362	0.372	0.360	0.914	0.455
	NABind*	0.632	0.330	0.405	0.400	0.909	0.464
	NABind	**0.658**	**0.432**	**0.484**	**0.481**	**0.937**	**0.550**
RBR_117	COACH-D*	0.167	0.107	0.116	0.093	0.568	0.147
	COACH-D	0.223	0.133	0.150	0.132	0.602	0.186
	NCBRPred	0.262	0.133	0.143	0.118	0.665	0.200
	NucBind*	0.543	0.155	0.219	0.180	0.721	0.230
	NucBind	0.407	0.204	0.238	0.200	0.725	0.243
	SVMnuc	0.406	0.202	0.237	0.192	0.725	0.241
	aaRNA*	0.339	0.208	0.221	0.172	0.725	0.239
	aaRNA	0.475	0.209	0.249	0.206	0.759	0.267
	NucleicNet*	0.405	0.181	0.222	0.174	0.660	0.198
	NucleicNet	0.461	0.286	0.315	0.286	0.740	0.306
	PSTPRNA*	0.544	0.198	0.259	0.224	0.763	0.274
	PSTPRNA	0.644	0.222	0.293	0.278	0.809	0.343
	RBRDetector*	0.527	0.232	0.277	0.248	0.790	0.318
	RBRDetector	0.545	0.274	0.313	0.291	0.818	0.359
	GraphBind*	0.470	0.224	0.257	0.229	0.780	0.285
	GraphBind	0.536	0.305	0.324	0.312	0.848	0.386
	NABind*	0.546	0.280	0.330	0.311	0.852	0.383
	NABind	**0.659**	**0.306**	**0.377**	**0.368**	**0.882**	**0.457**
RBR_106	COACH-D*	0.215	0.132	0.152	0.127	0.598	0.209
	COACH-D	0.235	0.185	0.189	0.162	0.618	0.239
	NCBRPred	0.233	0.224	0.171	0.145	0.673	0.255
	NucBind*	0.515	0.209	0.277	0.211	0.718	0.275
	NucBind	0.439	0.223	0.276	0.207	0.717	0.275
	SVMnuc	0.546	0.212	0.285	0.213	0.728	0.276
	aaRNA*	0.432	0.236	0.280	0.206	0.727	0.277
	aaRNA	0.425	0.275	0.303	0.238	0.762	0.324
	NucleicNet*	0.367	0.237	0.265	0.193	0.647	0.231
	NucleicNet	0.501	0.327	0.365	0.314	0.728	0.353
	PSTPRNA*	0.341	0.118	0.164	0.082	0.589	0.173
	PSTPRNA	0.627	0.334	0.406	0.363	0.840	0.418
	RBRDetector*	0.549	0.254	0.313	0.254	0.771	0.339
	RBRDetector	0.387	0.363	0.334	0.288	0.796	0.380
	GraphBind*	0.412	0.284	0.294	0.243	0.748	0.325
	GraphBind	0.489	0.379	0.378	0.341	0.818	0.436
	NABind*	0.507	0.391	0.393	0.357	0.854	0.452
	NABind	**0.638**	**0.443**	**0.479**	**0.449**	**0.884**	**0.542**

* represents trRosetta-based predicted protein structures used for evaluation.

### Case studies

**Fig 5:**
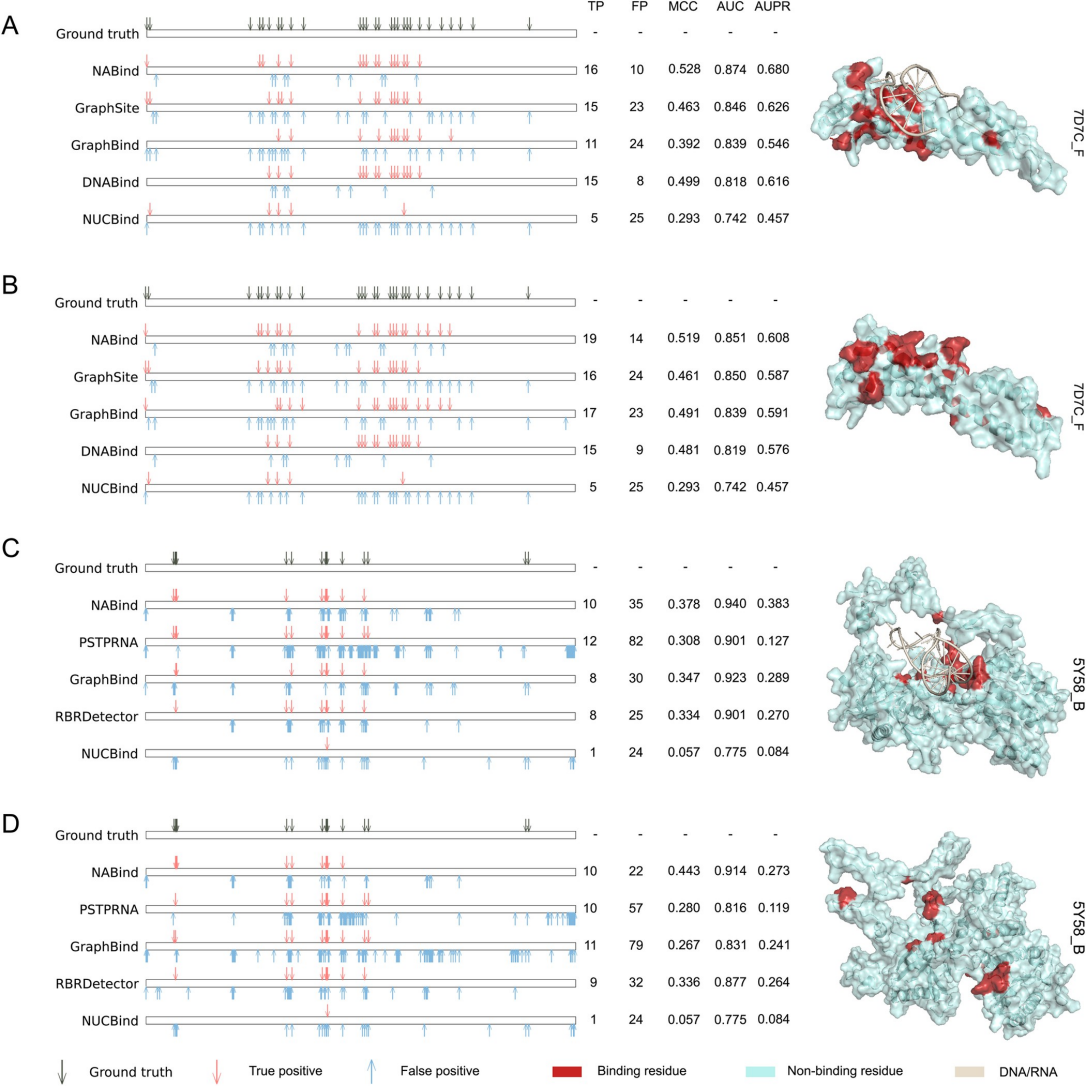
Prediction results of several examples generated by NABind and other state-of-the-art methods. (A) Results for the native structure of a DBP (PDB ID: 7D7C_F). (B) Results for the trRosetta-based predicted structure of this DBP. (C) Results for the native structure of an RBP (PDB ID: 5Y58_B). (D) Results for the trRosetta-based predicted structure of this RBP.

From the DBR_181 and RBR_117 datasets, we selected two examples for case studies and displayed the prediction results of different methods. Fig 5A shows the complex of a DBP (PDB ID: 7D7C_F) and its ligand DNA. This protein has a total of 137 residues, of which 28 are binding residues. NABind obtained the highest measures (MCC: 0.528, AUC: 0.874, and AUPR: 0.680) and achieved 16 true positives among 26 predicted binding residues. Despite yielding a comparable number of true positives, both GraphSite and GraphBind had approximately twice as many false positives as NABind, resulting in their worse performance. For the predicted structure of this protein, the performance of GraphSite was relatively stable, while GraphBind whose performance was strengthened recovered 17 binding residues (Fig 5B). By generating more true positives and fewer false positives, NABind obtained better results than the competing methods (MCC: 0.519, AUC: 0.851, and AUPR: 0.608). Fig 5C and 5D shows the prediction results for the native and predicted structures of an RBP (PDB ID: 5Y58_B). This protein includes 568 residues, among which 15 are binding residues. For the native structure, NABind correctly predicted 10 binding residues, which was slightly less than the measure of PSTPRNA (12 true positives). However, the false positives of PSTPRNA were approximately two and a half times those of NABind. For the predicted structure, PSTPRNA was still inferior to our method due to its greater number of false positives. Although GraphBind achieved the largest number of true positives in this case, the greatest number of false positives also resulted in its ordinary performance. In contrast, NABind yielded remarkably fewer false positives and showed the best performance on both the native and predicted structures (MCC: 0.378 and 0.443, AUC: 0.940 and 0.914, and AUPR: 0.383 and 0.273).

## Discussion

The primary innovation of our algorithm is the combination of deep learning- and template-based methods for DBR and RBR predictions. NABind achieved excellent performance on both native and predicted structures, which could be attributed to the interplay among our four basic modules. In the deep learning module, embeddings from protein language models and novel structural features were applied to edge-featured graph attention networks. Results showed that sequence-based features were more effective than structure-based counterparts, and the integration of them could lead to more favorable prediction accuracy. Moreover, the structural context learned by EGAT was superior to the traditional residue representation. Although the deep learning module played a major role in our algorithm, the improved template method provided complementary information by using relevant features extracted from multiple high-quality templates for supervised learning. Accordingly, when the two modules were merged through a stacking strategy, we observed a beneficial effect on the overall performance. The correction of the integrative results by the post-processing module further improved the prediction accuracy. Because of the contribution of each module, NABind not only outperformed conventional hybrid algorithms by a large margin but also surpassed recent deep learning-based approaches.

Although the progress was achieved, our algorithm still had several limitations. We noticed that the utility of our template-based module remarkably decreased when NABind was applied to predicted protein structures, indicating that this module was very sensitive to structural changes. Therefore, the robustness of the template-based method should be enhanced. Because DNA- and RNA-binding regions share many similarities, most existing predictors of DBRs could cross-predict many RBRs as DNA-binding, and vice versa. In other words, the discrimination between DBRs and RBRs is difficult. To check whether our method suffered from this issue, the cross-prediction was performed by training the model on the DBR (RBR) set and testing the model on the RBR (DBR) set. As seen in S8 Table, the prediction results were very close to those of canonical tests, probably due to the similar binding patterns shared by DBRs and RBRs (S2 Fig). Accordingly, we need to design specific features for discriminating between these two types of binding residues. In conclusion, we present NABind, an accurate algorithm for predicting nucleic acid binding residues, and the proposed strategies could be applied to other types of protein functional sites in the future.

## Materials and methods

### Dataset collection

Here, we derived three DBR datasets and two RBR datasets from previous studies. Based on the number of proteins, the five datasets were named DBR_573, DBR_129, DBR_181, RBR_495 and RBR_117, respectively (S9 Table). Among these datasets, DBR_181 was collected by GraphSite, and the other four datasets were prepared by GraphBind. This could be beneficial to a fair comparison of our method and other state-of-the-art deep learning models [14,15]. As suggested by previous works, DBR_573 and RBR_495 were utilized as the training sets to develop DBR- and RBR-related prediction models, respectively, and the remaining datasets were treated as the corresponding test sets. Note that the sequence identities within the training set and those between the training and test sets were less than 30% by using CD-HIT. All the entries in these datasets were collected from the BioLiP database [21]. A residue was considered to be a binding residue if the distance between any atom of this residue and any atom of the nucleic acid molecule was less than 0.5 Å plus the van der Waal’s radius of these two atoms. As suggested by GraphBind, the data augmentation procedure was applied to the training sets to alleviate the data imbalance problem of binding and non-binding residues [14]. Briefly, the creators of DBR_573 and RBR_495 transferred the binding annotations from other protein chains in BioLiP with high sequence and structural similarities (sequence identity > 0.8 and TMscore > 0.5) to the representative chain in these two datasets. As a result, the percentages of DBRs and RBRs were increased by 30.7% and 24.3%, respectively. Additionally, based on the latest BioLiP database (version: 2023-04-13), we constructed another non-redundant test set of 106 RBPs (termed RBR_106). The sequence identities between RBR_106 and the other two RBR-related datasets were less than 30%. For each dataset, in addition to the native structure of proteins, the predicted structures were generated by trRosetta. Regarding the test sets, AlphaFold2-based predicted structures for DBPs were provided by GraphSite, while those for RBPs were retrieved from the AlphaFold database (S1 Text) [15,22,23].

### Feature extraction

#### Sequence-related features

*MSA embedding*. Trained on a huge number of single sequences or multiple sequence alignments (MSA), unsupervised protein language models are able to learn the structure and function of proteins [24–26]. Among existing protein language models, the ESM-MSA-1b, which takes an MSA as input and adopts row and column attentions, has shown excellent performance on various protein-related tasks [26]. The MSA embedding of each sequence was generated using ESM-MSA-1b with the input of 128 sequences achieving the maximum Hamming distance in an MSA.

*Position specific scoring matrix*. To yield the position specific scoring matrix (PSSM), each sequence was searched against the UniRef90 database using PSI-BLAST with three iterations and an E-value of 0.001 [27]. The values in the matrix were normalized using the sigmoid function as follows:

$$y=\frac{1}{1+e^{-x}}$$

*Hidden Markov models profile*. Different from PSI-BLAST, HHblits utilizes Hidden Markov Models (HMM) to find remote homologs [28]. The HMM profile was generated using HHblits with default parameters to search against the UniClust30 database. The original profile was composed of emission frequencies, and the inverse transformation was thus conducted as follows:

$$y=2^{-0.001x}$$

#### Structure-related features

*Structural embedding*. ESMIF, one of the state-of-the-art algorithms for protein design, is adopted to recover sequences from backbone structures [29]. In this study, the structural embedding of each residue was translated from the backbone structure by ESMIF for the prediction of binding residues.

*Graph-based descriptors*. Each protein can be represented as a graph, where residues are treated as nodes and the edges are identified according to our previous studies [19,20]. As suggested by Cha *et al*.’s work, the Ollivier Ricci curvature (ORC) and Forman Ricci curvature (FRC) were used to measure the geodesic path complexity of nodes based on Riemannian geometry [30–33]. ORC could be interpreted by the optimal transport theory, in which edges with positive measures form communities, whereas edges with negative measures are treated as bridges between communities. The formula can be presented as follows:

$$ORC(x)=\sum_{y\in e(x,y)}{ORC}_{e}(x,y)=\sum_{y\in e(x,y)}1-\frac{W(m_{x},m_{y})}{d(x,y)}$$

where *e*(*x*, *y*) is the set of edges formed by nodes *x* and *y*, *m*_*x*_ and *m*_*y*_ are density distributions, and *W*(*m*_*x*_, *m*_*y*_) is the Wasserstein distance used to measure the minimum cost for transforming *m*_*x*_ into *m*_*y*_. Unlike ORC, FRC is dependent on the framework of CW complexes and can be calculated as follows:

$$FRC(x)=\sum_{y\in e(x,y)}{FRC}_{e}(x,y)=\sum_{y\in e(x,y)}w_{e}(\frac{w_{x}}{w_{e}}+\frac{w_{y}}{w_{e}}-\sum_{x,y\in e(x,y)}\left[\frac{w_{x}}{\sqrt{w_{e}w_{ex}}}+\frac{w_{y}}{\sqrt{w_{e}w_{ey}}}\right])$$

where *w*_*e*_, *w*_*x*_ and *w*_*y*_ are the weights of edge *e*, node *x* and node *y*. *w*_*ex*_ and *w*_*ey*_ are the weights of edge *e* incident on nodes *x* and *y*. Edge-based curvatures, namely *ORC*_*e*_(*x*, *y*) and *FRC*_*e*_(*x*, *y*), were computed using the *GraphRicciCurvature* python package.

Besides, the node-based multifractal dimension (MFD) is an indicator to measure the network complexity in multifractal analysis [30,34,35]. A higher MFD value indicates a higher degree of complexity. Using the box-covering method, we estimated this measure by investigating the power-law behavior between the mass distribution and the size of the box centered on the target node as follows:

$$y=\log\left(\frac{M(l)}{l}\right)$$

where *l* is the maximum value of the shortest paths between the target node and other nodes in the box, and *M*(*l*) is the mass distribution that is the number of nodes in the box. The graph-based descriptors were transformed into Z-scores based on the mean and standard deviation of all samples in the training set.

*Geometry-based descriptors*. Three local structural descriptors, including the minimum inaccessible radius, accessible shell volume and pocketness, were used to evaluate the extent of depth and exposure of a given residue. These measures were generated by the pocket detection software called GHECOM [36–38]. The geometry-based descriptors were also converted into Z-scores.

*Distance-based descriptors*. Referring to Xia *et al*.^’^s work, we adopted the distance-based features that measure the relative position of a residue in the protein structure at different scales [39]. To this end, the centroids of different fragments in the protein were treated as reference points, and the distances from the target residue to reference points were calculated as features. A smaller distance implies that the residue is closer to the center of a given context. The number of desired fragments determines the dimension of the feature vector, which could be presented as follows:

$$P_{ref}=\frac{2^{m}}{N}\sum_{v\in V_{\left[(g-1)\frac{N}{2^{m}}]:[g\frac{N}{2^{m}}\right]}}v\;g\in \{1,2,\ldots 2^{m}\}$$

$$y=\|t-p\|\;p\in P_{ref}$$

where 2^*m*^ (*m*∈{0,1,2}) represents the number of desired fragments, *N* represents the sequence length, $V_{\left[(g-1)\frac{N}{2^{m}}]:[g\frac{N}{2^{m}}\right]}$ represents a contiguous fragment of the protein, and *t* represents the coordinates of target residue.

Additionally, the distance descriptors that are dependent on the ultrafast shape recognition (USR) method were used to capture the topological relationship between the target residue and the global structure [40]. The topological properties of each residue could be characterized by the distance sets relevant to three reference residues, namely the target residue *r*, the residue *b* that is farthest from *r*, and the residue *g* that is farthest from *b*. Thus, the USR features can be calculated as follows:

$$\left\{ \begin{array}{c} u^{r}=\frac{1}{L}\sum_{1\leq i\leq L}\left||r-a_{i}|\right|\\ u^{b}=\frac{1}{L}\sum_{1\leq i\leq L}\left||b-a_{i}|\right|\\ u^{g}=\frac{1}{L}\sum_{1\leq i\leq L}\left||g-a_{i}|\right| \end{array}\right.$$

where *L* is the sequence length and *a*_*i*_ is the coordinates of the *i*th residue.

*Distance and orientation between residues*. The distance between two residues is defined as the shortest distance between their heavy atoms. The relative orientation between residues is defined as the absolute value of the angle between the surface normals of the planes for the two residues, and the plane is determined by the alpha carbon atom, the carbon atom of the carboxyl group, and the nitrogen atom of the amino group. These two descriptors were used as edge features in EGAT.

#### Template-related features

Based on each query-template pair, the overall alignment features and the residue-based alignment features were calculated. The first category included the sequence identity between the query and template proteins provided by NWalign and the structure alignment score output by TMalign [41]. The second category included the BLOSUM62 alignment score, secondary structure alignment score (S11 Table), distance between aligned residues, and binding probability [42–44]. To achieve the last two features, the query protein was superimposed onto the template structure. If the target residue was within 4.5 Å of the nucleic acids, its binding probability was 1; otherwise, the binding probability was 0 [19,20].

### Deep learning-based module

The protein structure was represented as a graph in which the nodes are residues and the edges indicate a contact between two residues if the distance between any atoms of the residues is less than 10Å. As shown in Fig 1B, subsequently, a variety of features were extracted and assigned to nodes and edges. Edge features included the distance between two residues and the relative orientation of two residues, while node features included all the sequence- and structure-related descriptors mentioned above. The constructed graph was fed into the graph neural network, which could update the representation of each residue. EGAT, an extension of the graph attention network (GAT) by incorporating edge features, was adopted to implement the graph neural network [45]. GAT computes the attention scores mainly based on node features, whereas EGAT estimates the weights using both node and edge features. Given the initial feature *h*_*i*_, the update of node i can be presented as follows:

$$h_{i}^{\prime }=Wh_{i}+b$$

where $h_{i}^{\prime }$ is the updated node feature, *W* is the parameter matrix, and *b* is the bias. The attention score was derived from the formula:

$$a_{ij}=\frac{exp(e_{ij})}{\sum_{k\in N_{i}}exp(e_{ik})}$$

where *a*_*ij*_ denotes the attention score, and *N*_*i*_ denotes the neighbors of node i. *e*_*ij*_ is the unnormalized weight, which was calculated by the following equations:

$$e_{ij}=Ff_{ij}^{\prime }$$

$$f_{ij}^{\prime }=LeakyReLU(A[h_{i}^{\prime }||f_{ij}||h_{j}^{\prime }])$$

where *f*_*ij*_ and $f_{ij}^{\prime }$ denote the edge features before and after the update, respectively. *F* and *A* are learnable parameter matrices. The node messages output from the EGAT layer were calculated as follows:

$$h_{i}^{\prime \prime }=\sum_{k\in N_{i}}a_{ik}h_{k}^{\prime }$$

In this work, the node features derived from the last layer together with the initial node features were passed through the fully connected layer to yield the binding probabilities:

$$Y=Sigmoid(W_{2}(ReLU(W_{1}[H\|H^{\prime \prime }]+b_{1}))+b_{2})$$

where *W*_1_, *W*_2_, *b*_1_ and *b*_2_ are learnable parameters. *H* and *H*′′ are the original and updated node features, respectively. This framework included one EGAT layer and one fully connected layer. We combined PyTorch and DGL to implement the prediction model, which was trained for 50 epochs [46,47]. The loss function was the weighted cross-entropy loss in which the weight of positive samples was set to 50 [48]. The Nesterov accelerated gradient was used in the model with the momentum factor of 0.9, the L2 penalty of 1e-6, and the learning rate of 1e-4. To avoid overfitting, dropout with a ratio of 0.5 was applied to the fully connected layer [49]. By choosing different random seeds, we trained five models to enhance the robustness of our prediction results.

### Template-based module

To construct the template library, we retrieved all entries with the ligand ID of NUC from the BioLiP database [21]. Then, we classified these entries into DBP- and RBP-related libraries using an in-house script. After scanning a query structure against the template library, we eliminated structurally irrelevant alignments and highly similar structures. Accordingly, reserved templates should satisfy the following constraints: the TMscore was greater than 0.3, and the sequence identity was less than 0.3 [41]. Considering the sequence redundancy among the retained templates, we further divided them into different clusters using CD-HIT with 90% sequence identity [50]. In each cluster, the template with the highest TMscore was chosen. These representatives were ranked according to their TMscores, and the top 20 templates were finally reserved. For each residue in the query, template-related features were generated based on the selected templates (see Feature extraction). If the number of templates was less than 20, the values of corresponding features were set to 0. Based on these features, three classifiers were trained to identify binding residues using the random forest, extreme gradient boosting and light gradient boosting machine [51–53].

### Merging module

To fully make use of the interplay between the deep learning and template modules, we constructed a meta-classifier by using a stacked generalization strategy [54]. Briefly, the five deep learning-based classifiers and the three template-based classifiers were treated as lower-level models, and their prediction scores for each residue were used as the input features of a light gradient boosting machine, which served as the higher-level model and generated the integrative predictions. All the hyper-parameters of classifiers are summarized in S12 Table.

### Post-processing module

Inspired by our previous work for predicting binding sites in nucleic acids, we developed a post-processing module based on the random walk with restart (RWR) algorithm [55]. Note that only protein surface residues were involved in this procedure. To this end, the protein surface should be converted into a residue network. The RWR is a diffusion process that allows information (i.e. binding probabilities) to be transferred between residues in the network, so that the binding probabilities of residues could be repeatedly optimized until the network achieves a stable state. The weight matrix *W* representing the probability of moving from residues to their neighbors was calculated as follows:

$$w_{ij}=\left\{ \begin{array}{l} \frac{\delta -d_{ij}}{\delta }\;if\;d_{ij}<\delta ,{rsa}_{i}>0.1\;and\;{rsa}_{j}>0.1\\ 0\;otherwise \end{array}\right.$$

where *δ* is the distance cutoff for generating residue networks (i.e. 10 Å), *d*_*ij*_ is the distance between residues *i* and *j*, and *rsa*_*i*_and *rsa*_*j*_ are the relative solvent accessibility of residues *i* and *j*, which were calculated by NACCESS [56]. The distance threshold is identical to the constraint of graph representations in our deep learning module, while the *rsa* cutoff is derived from our previous algorithms. The column-wise normalization was conducted on this matrix, and then the RWR algorithm was implemented as follows:

$$p_{t+1}=(1-\beta )p_{t}W+\beta p_{0}$$

where *p*_*t*+1_ and *p*_*t*_ denote the probability vector of surface residues at *t*+1 and *t* steps, respectively. *p*_0_ denotes the initial probability vector which is the result of the merging module. *β* is the restart probability which denotes the probability of the walker going back to the source node and was set to 0.9. When |*p*_*t*+1_−*p*_*t*_|<1*e*−2, the RWR process was terminated and *p*_*t*_ was seen as the final probability vector.

### Performance evaluation

We performed 5-fold cross-validation on the training sets and independent testing on the test sets. Six widely used measures were adopted to evaluate the effectiveness of the proposed model. Recall, precision, F1 and Matthews correlation coefficient (MCC) were calculated as follows:

$$Recall=\frac{TP}{TP+FN}$$

$$Precision=\frac{TP}{TP+FP}$$

$$F1=\frac{2*Recall*Precision}{Recall+Precision}$$

$$MCC=\frac{TP\times TN-FP\times FN}{\sqrt{\left(TP+FN)(TP+FP)(TN+FN)(TN+FP\right)}}$$

where TP, TN, FP, and FN are the number of true positives, true negatives, false positives, and false negatives, respectively. In addition, the area under the receiver operating characteristic curve (AUC) and area under the precision-recall curve (AUPR) were calculated. The MCC, AUC and AUPR were used as major measures. Here, we used both protein- and residue-based evaluations. For the first strategy, the measures were calculated for each protein, and the average results were reported for each dataset. For the second strategy, the measures were computed by concatenating the predictions of all residues in each dataset. Because the first strategy is more rigorous, it was chosen as the major method. Notably, regarding the deep learning and template modules, the mean score of the outputs of multiple classifiers was applied to performance evaluation.

### Statistical test

Statistical tests were performed to evaluate significant differences in performance between different models. For a given dataset, 70% of the proteins were randomly selected, and the mean values of AUC and AUPR were calculated. This procedure was repeated ten times. The Anderson-Darling test was then used to assess whether these values obey normal distribution. Based on the normality assumption, the paired t-test or Wilcoxon rank sum test was selected for statistical tests.

## Supporting information

S1 Text Extraction of predicted structures for RBPs from AlphaFold database. (PDF)

S1 Fig Comparison of deep learning models using different types of features on training sets. (A) AUPR measures for different types of features. Statistical tests were performed as described in the Methods section. **** *p* < 0.0001, *** 0.0001 ≤ *p* < 0.001, ** 0.001 ≤ *p* < 0.01, * 0.01 ≤ *p* < 0.05 and ns: *p* ≥ 0.05. (B) Density curves of AUC and AUPR for native structures using different types of features. (C) Numbers of binding residues retrieved by different types of features for native structures and predicted structures (*). (D) ROC and precision-recall curves for native and predicted structures using different types of features.
(PDF)

S2 Fig Comparison of structural features of binding and non-binding residues in training sets. (A-C) Graph-based descriptors. (D-F) Geometry-based descriptors. (G-P) Distance-based descriptors. Significant differences were evaluated using Wilcoxon rank sum test. **** *p* < 0.0001, *** 0.0001 ≤ *p* < 0.001, ** 0.001 ≤ *p* < 0.01, * 0.01 ≤ *p* < 0.05 and ns: *p* ≥ 0.05.
(PDF)

S3 Fig Comparison of the performance between deep learning- and template-based modules on training sets. (A) Comparison of these two modules in terms of the AUC measure. (B) Comparison of these two modules in terms of the AUPR measure.
(PDF)

S4 Fig Utility of the post-processing module. (A) Comparison of true positives (TP), true negatives (TN), false positives (FP), and false negatives (FN) before and after post-processing. (B) An example chosen from the DBR_573 dataset (PDB ID: 4Z8F_H).
(PDF)

S5 Fig Effects on the performance of template-based module when replacing native structures with predicted structures. (A) Negative correlation between the similarity of native and predicted structures (TMscore) and the difference in AUC. (B) Predicted structures with low quality showing a remarkable decrease in AUC and AUPR.
(PDF)

S6 Fig Comparison of merging strategies in our previous and current algorithms (i.e. weighted combination and stacking, respectively). (A) Searching optimal parameters for previous methods on DBR_573. (B) Searching optimal parameters for previous methods on RBR_495. (C) Performance of these two strategies on DBR_573. (D) Performance of these two strategies on RBR_495.
(PDF)

S7 Fig Comparison of NABind^DL^ and the state-of-the-art deep learning methods on test sets. (A) Results for DBR_129. (B) Results for DBR_129*. (C) Results for DBR_181. (D) Results for DBR_181*. (E) Results for RBR_117. (F) Results for RBR_117*. (G) Results for RBR_106. (H) Results for RBR_106*. * represents trRosetta-based predicted structures used for evaluation.
(PDF)

S1 Table Performance of deep learning modules using different types of features. (PDF)

S2 Table P-values of performance difference between merging module and its two submodules. (PDF)

S3 Table Comparison of NABind and our previous methods on different datasets. (PDF)

S4 Table Residue-based performance of NABind and other methods on test sets. (PDF)

S5 Table Comparison of NABind and deep learning methods on test sets. (PDF)

S6 Table Residue-based performance of NABind and deep learning methods on test sets. (PDF)

S7 Table Performance of different modules on test sets. (PDF)

S8 Table Performance of cross-prediction for DNA- and RNA-binding residues. (PDF)

S9 Table A summary of datasets used in this study. (PDF)

S10 Table Sequence identity between structures from AlphaFold database and native structures. (PDF)

S11 Table Alignment scores of secondary structures. (PDF)

S12 Table Hyper-parameters for different modules. (PDF)
