# Supplementary material for: Structure-based prediction of nucleic acid binding residues by merging deep learning- and template-based approaches
Source: PLoS Comput Biol. 2023 Sep 6;19(9):e1011428. doi: 10.1371/journal.pcbi.1011428 (PMC10482303; doi:10.1371/journal.pcbi.1011428)
Supplement: S1 Text — (PDF) [file pcbi.1011428.s001.pdf]

S1 Text. Extraction of predicted structures for RBPs from AlphaFold database

We used a four-step method to retrieve AlphaFold2-based predicted structures for RBPs. First, we obtained the UniProt accession number corresponding to each PDB chain ID using SIFTS transformation. Second, we downloaded the corresponding predicted structures from the AlphaFold database based on UniProt accession number. Third, we aligned the sequence of the native structure with that of the above predicted structure using EMBOSS Needle. Fourth, we extracted the partitions corresponding to the native sequence from the predicted structures. As a result, 86 chains in RBR_117 and 64 chains in RBR_106 had AlphaFold2-based predicted structures. S10 Table provides the corresponding PDB IDs and sequence identities.
