# Supplementary material for: Structure-based prediction of nucleic acid binding residues by merging deep learning- and template-based approaches
Source: PLoS Comput Biol. 2023 Sep 6;19(9):e1011428. doi: 10.1371/journal.pcbi.1011428 (PMC10482303; doi:10.1371/journal.pcbi.1011428)
Supplement: S1 Fig — (A) AUPR measures for different types of features. Statistical tests were performed as described in the Methods section. **** p < 0.0001, *** 0.0001 ≤ p < 0.001, ** 0.001 ≤ p < 0.01, * 0.01 ≤ p < 0.05 and ns: p ≥ 0.05. (B) Density curves of AUC and AUPR for native structures using different types of features. (C) Numbers of binding residues retrieved by different types of features for native structures and predicted structures (*). (D) ROC and precision-recall curves for native and predicted structures using different types of features. (PDF) [file pcbi.1011428.s002.pdf]

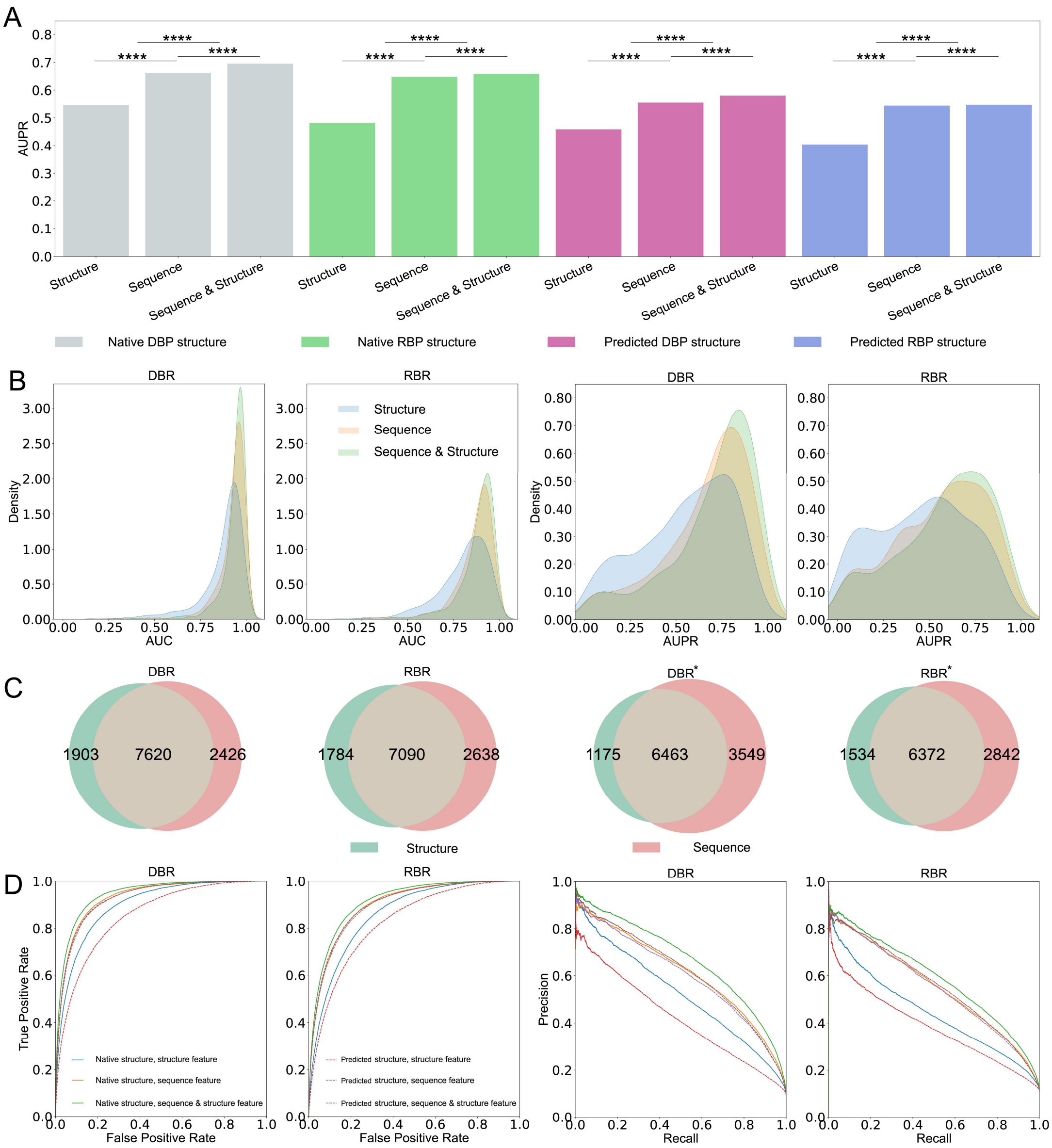

S1 Fig. Comparison of deep learning models using different types of features on training sets. (A) AUPR measures for different types of features. Statistical tests were performed as described in the Methods section. **** $p < 0.0001$, *** $0.0001 \leq p < 0.001$, ** $0.001 \leq p < 0.01$, * $0.01 \leq p < 0.05$ and ns: $p \geq 0.05$. (B) Density curves of AUC and AUPR for native structures using different types of features. (C) Numbers of binding residues retrieved by different types of features for native structures and predicted structures (*). (D) ROC and precision-recall curves for native and predicted structures using different types of features.
