# Supplementary material for: Structure-based prediction of nucleic acid binding residues by merging deep learning- and template-based approaches
Source: PLoS Comput Biol. 2023 Sep 6;19(9):e1011428. doi: 10.1371/journal.pcbi.1011428 (PMC10482303; doi:10.1371/journal.pcbi.1011428)
Supplement: S2 Fig — (A-C) Graph-based descriptors. (D-F) Geometry-based descriptors. (G-P) Distance-based descriptors. Significant differences were evaluated using Wilcoxon rank sum test. **** p < 0.0001, *** 0.0001 ≤ p < 0.001, ** 0.001 ≤ p < 0.01, * 0.01 ≤ p < 0.05 and ns: p ≥ 0.05. (PDF) [file pcbi.1011428.s003.pdf]

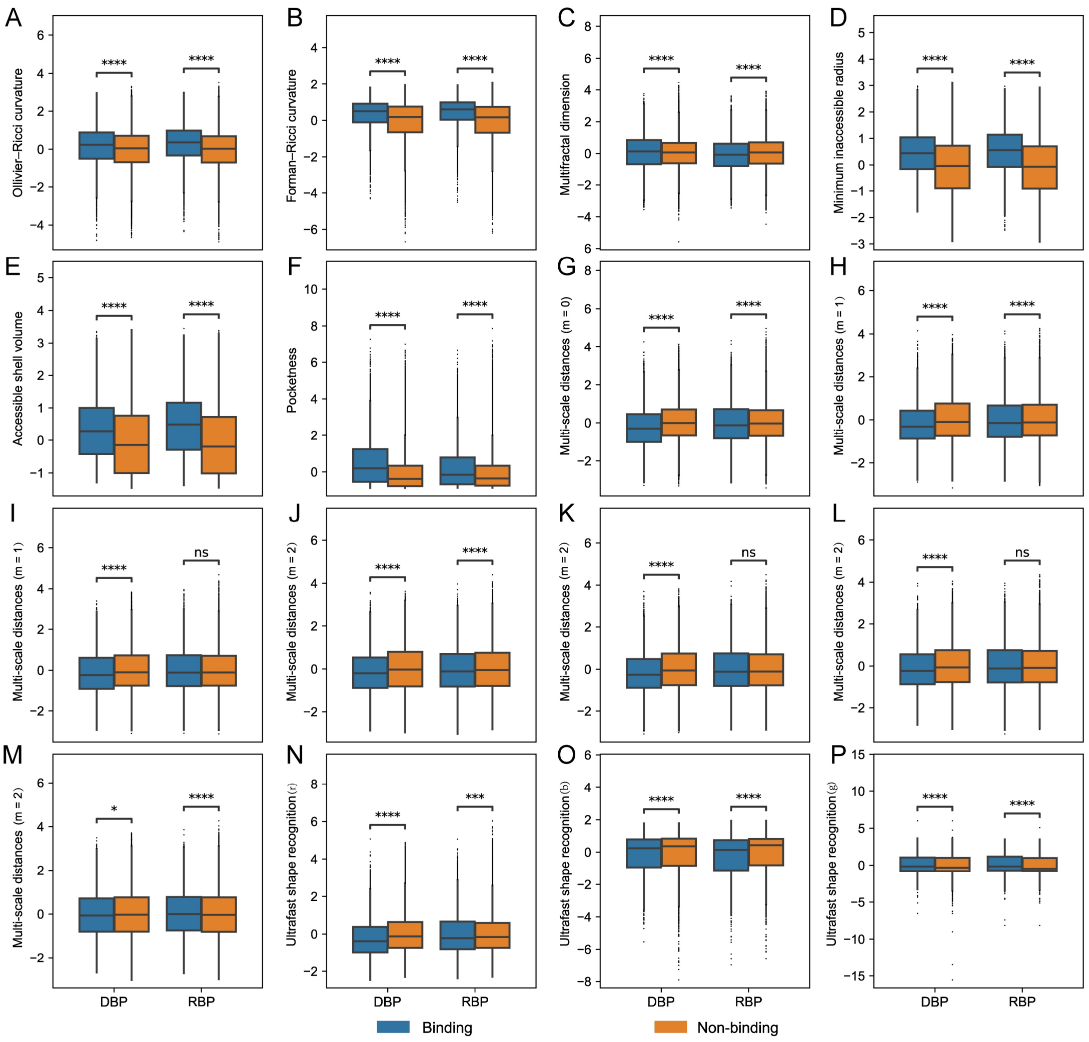

S2 Fig. Comparison of structural features of binding and non-binding residues in training sets. (A-C) Graph-based descriptors. (D-F) Geometry-based descriptors. (G-P) Distance-based descriptors. Significant differences were evaluated using Wilcoxon rank sum test. **** $p < 0.0001$, *** $0.0001 \le p < 0.001$, ** $0.001 \le p < 0.01$, * $0.01 \le p < 0.05$ and ns: $p \ge 0.05$.
