# Supplementary material for: Structure-based prediction of nucleic acid binding residues by merging deep learning- and template-based approaches
Source: PLoS Comput Biol. 2023 Sep 6;19(9):e1011428. doi: 10.1371/journal.pcbi.1011428 (PMC10482303; doi:10.1371/journal.pcbi.1011428)
Supplement: S3 Fig — (A) Comparison of these two modules in terms of the AUC measure. (B) Comparison of these two modules in terms of the AUPR measure. (PDF) [file pcbi.1011428.s004.pdf]

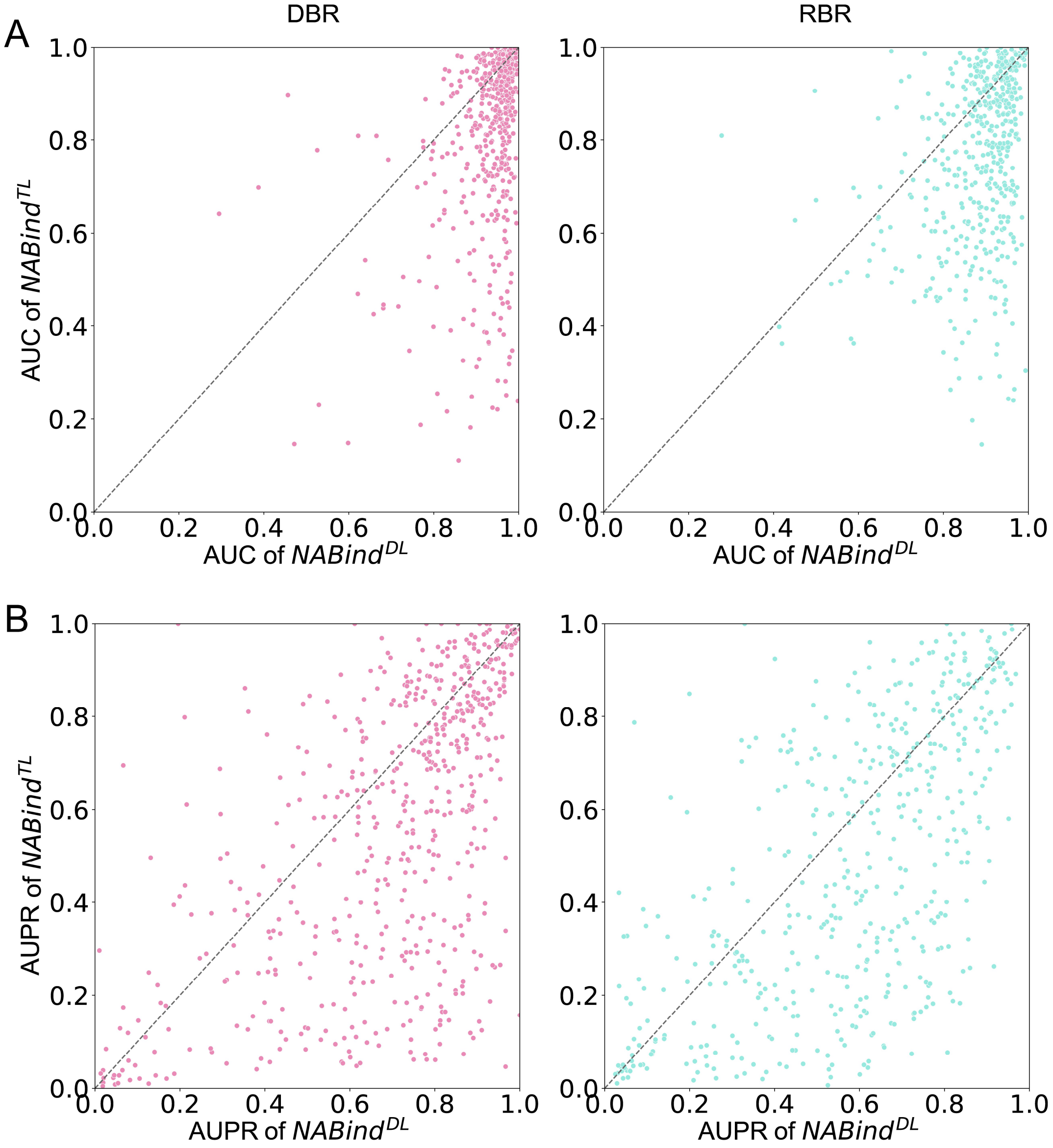

S3 Fig. Comparison of the performance between deep learning- and template-based modules on training sets. (A) Comparison of these two modules in terms of the AUC measure. (B) Comparison of these two modules in terms of the AUPR measure.
