# Supplementary material for: Structure-based prediction of nucleic acid binding residues by merging deep learning- and template-based approaches
Source: PLoS Comput Biol. 2023 Sep 6;19(9):e1011428. doi: 10.1371/journal.pcbi.1011428 (PMC10482303; doi:10.1371/journal.pcbi.1011428)
Supplement: S4 Fig — (A) Comparison of true positives (TP), true negatives (TN), false positives (FP), and false negatives (FN) before and after post-processing. (B) An example chosen from the DBR_573 dataset (PDB ID: 4Z8F_H). (PDF) [file pcbi.1011428.s005.pdf]

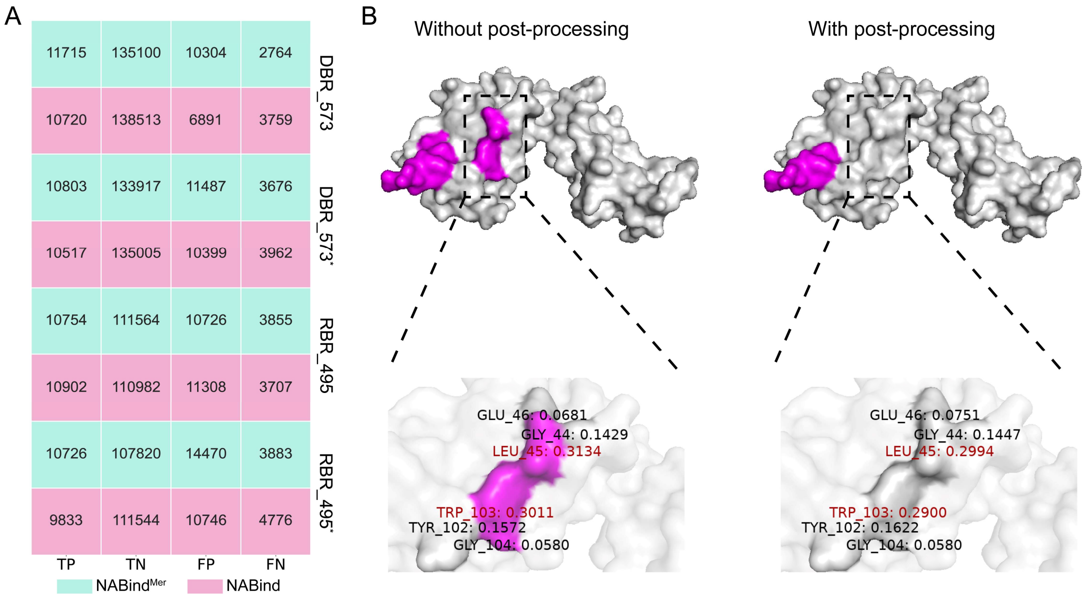

S4 Fig. Utility of the post-processing module. (A) Comparison of true positives (TP), true negatives (TN), false positives (FP), and false negatives (FN) before and after post-processing. (B) An example chosen from the DBR_573 dataset (PDB ID: 4Z8F_H). The predicted binding residues are highlighted in magenta in the structure. Without the post-processing, 6 out of 9 positive predictions were false positives. The cutoff of binding probability was 0.3. The false positive residues LEU_45 and TRP_103 (zoomed region) were successfully removed by post-processing.
