# Supplementary material for: Structure-based prediction of nucleic acid binding residues by merging deep learning- and template-based approaches
Source: PLoS Comput Biol. 2023 Sep 6;19(9):e1011428. doi: 10.1371/journal.pcbi.1011428 (PMC10482303; doi:10.1371/journal.pcbi.1011428)
Supplement: S5 Fig — (A) Negative correlation between the similarity of native and predicted structures (TMscore) and the difference in AUC. (B) Predicted structures with low quality showing a remarkable decrease in AUC and AUPR. (PDF) [file pcbi.1011428.s006.pdf]

A

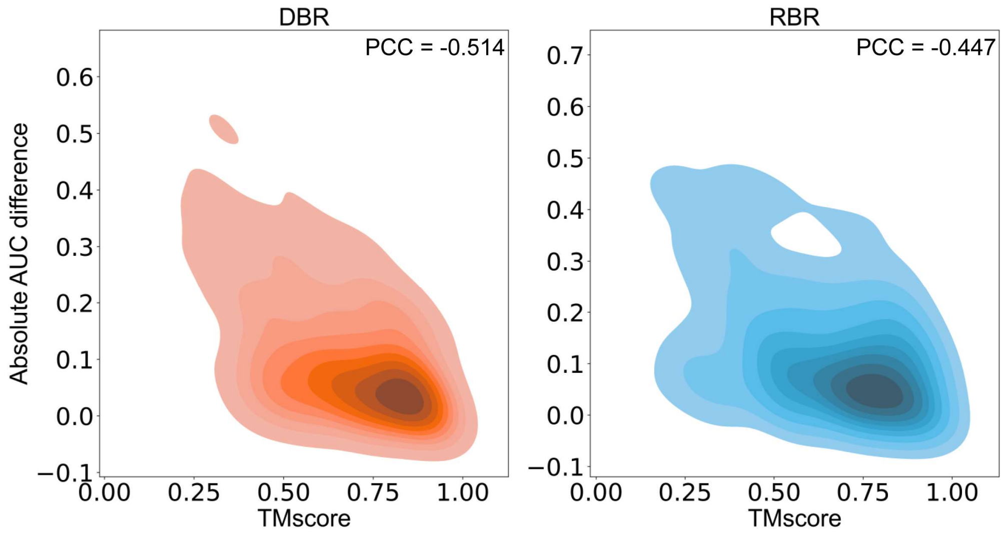

B

Dataset	DBR_573			RBR_495		
	Number of proteins	Decrease in AUC	Decrease in AUPR	Number of proteins	Decrease in AUC	Decrease in AUPR
TMscore>0.5	474	0.037	0.078	410	0.032	0.062
0.2≤TMscore≤0.5	95	0.147	0.187	81	0.124	0.137
TMscore<0.2	4	0.213	0.218	4	0.355	0.339

S5 Fig. Effects on the performance of template-based module when replacing native structures with predicted structures. (A) Negative correlation between the similarity of native and predicted structures (TMscore) and the difference in AUC. (B) Predicted structures with low quality showing a remarkable decrease in AUC and AUPR.
