# Supplementary material for: Structure-based prediction of nucleic acid binding residues by merging deep learning- and template-based approaches
Source: PLoS Comput Biol. 2023 Sep 6;19(9):e1011428. doi: 10.1371/journal.pcbi.1011428 (PMC10482303; doi:10.1371/journal.pcbi.1011428)
Supplement: S6 Fig — (A) Searching optimal parameters for previous methods on DBR_573. (B) Searching optimal parameters for previous methods on RBR_495. (C) Performance of these two strategies on DBR_573. (D) Performance of these two strategies on RBR_495. (PDF) [file pcbi.1011428.s007.pdf]

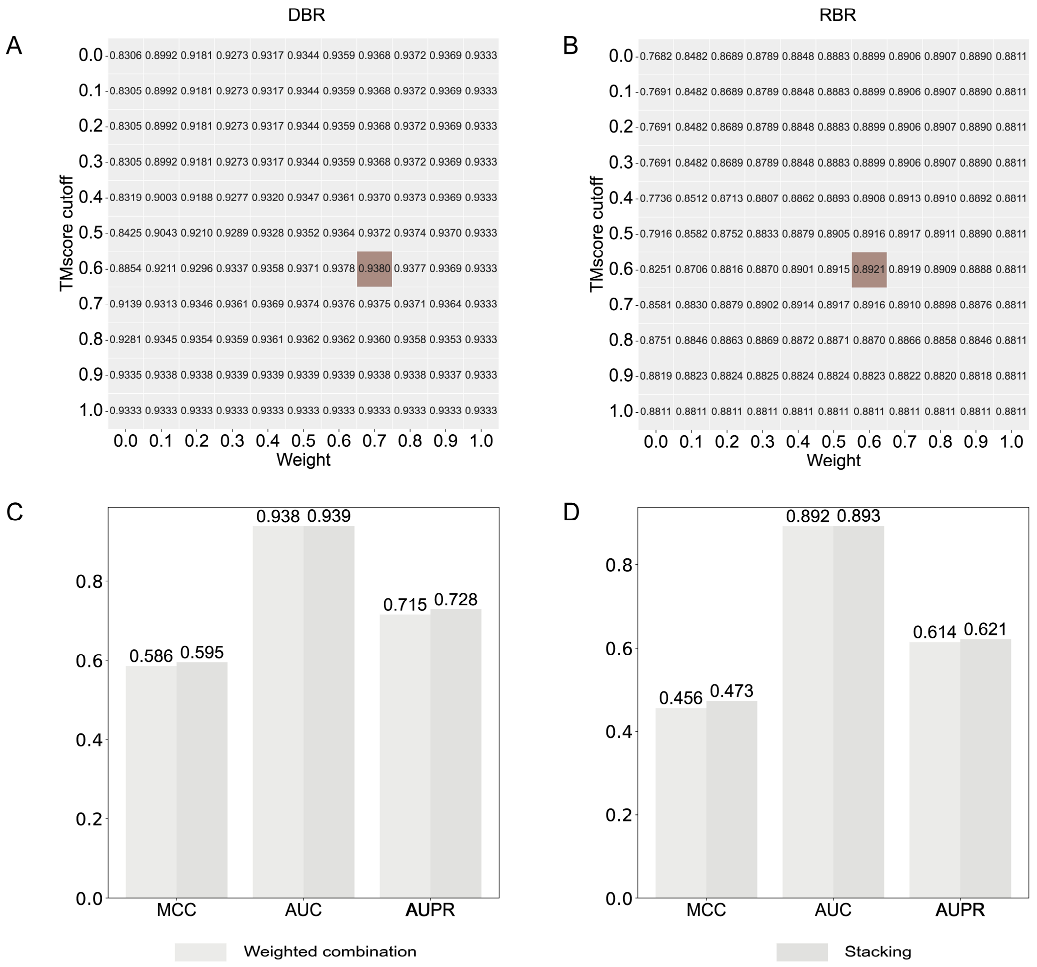

S6 Fig. Comparison of merging strategies in our previous and current algorithms (i.e. weighted combination and stacking, respectively). (A) Searching optimal parameters for previous methods on DBR_573. (B) Searching optimal parameters for previous methods on RBR_495. (C) Performance of these two strategies on DBR_573. (D) Performance of these two strategies on RBR_495. The strategy used previously is shown as follows:

$$\begin{cases} Score = DLscore \times Weight + TLscore \times (1 - Weight) & if\ TMscore > cutoff \\ Score = DLscore & if\ TMscore \leq cutoff \end{cases}$$

where $Score$, $DLscore$, and $TLscore$ denote the binding probabilities generated by the merging module, deep learning module, and template module, respectively.
