# Supplementary material for: Structure-based prediction of nucleic acid binding residues by merging deep learning- and template-based approaches
Source: PLoS Comput Biol. 2023 Sep 6;19(9):e1011428. doi: 10.1371/journal.pcbi.1011428 (PMC10482303; doi:10.1371/journal.pcbi.1011428)
Supplement: S7 Fig — (A) Results for DBR_129. (B) Results for DBR_129*. (C) Results for DBR_181. (D) Results for DBR_181*. (E) Results for RBR_117. (F) Results for RBR_117*. (G) Results for RBR_106. (H) Results for RBR_106*. * represents trRosetta-based predicted structures used for evaluation. (PDF) [file pcbi.1011428.s008.pdf]

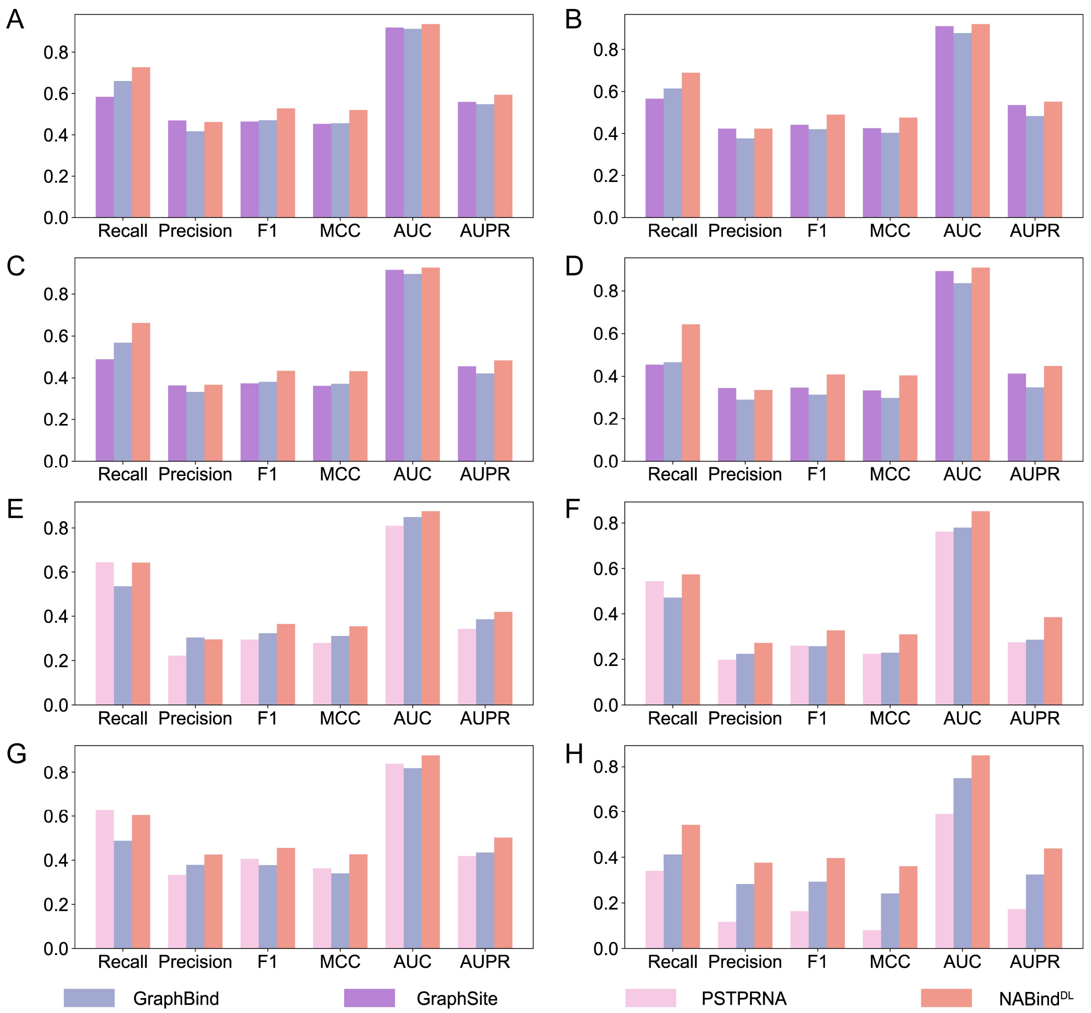

S7 Fig. Comparison of NABind^{DL} and the state-of-the-art deep learning methods on test sets. (A) Results for DBR_129. (B) Results for DBR_129*. (C) Results for DBR_181. (D) Results for DBR_181*. (E) Results for RBR_117. (F) Results for RBR_117*. (G) Results for RBR_106. (H) Results for RBR_106*. * represents trRosetta-based predicted structures used for evaluation.
