# Supplementary material for: Structure-based prediction of nucleic acid binding residues by merging deep learning- and template-based approaches
Source: PLoS Comput Biol. 2023 Sep 6;19(9):e1011428. doi: 10.1371/journal.pcbi.1011428 (PMC10482303; doi:10.1371/journal.pcbi.1011428)
Supplement: S1 Table — (PDF) [file pcbi.1011428.s009.pdf]

S1 Table. Performance of deep learning modules using different types of features

Dataset	Feature	Recall	Precision	F1	MCC	AUC	AUPR
DBR_573	PSSM & HHM	0.560	0.396	0.416	0.361	0.831	0.464
	ESM-MSA-1b	0.709	0.530	0.577	0.541	0.919	0.655
	Sequence	0.711	0.540	0.583	0.548	0.920	0.661
	ESMIF	0.586	0.440	0.474	0.421	0.862	0.528
	Structure	0.651	0.426	0.488	0.437	0.871	0.548
	Sequence & structure	0.748	0.549	0.606	0.575	0.931	0.695
DBR_573*	PSSM & HHM	0.614	0.376	0.426	0.366	0.826	0.455
	ESM-MSA-1b	0.704	0.519	0.570	0.531	0.914	0.642
	Sequence	0.734	0.511	0.574	0.537	0.915	0.647
	ESMIF	0.572	0.363	0.417	0.351	0.822	0.459
	Structure	0.584	0.382	0.434	0.371	0.834	0.482
	Sequence & structure	0.713	0.532	0.581	0.545	0.918	0.658
RBR_495	PSSM & HHM	0.544	0.335	0.371	0.261	0.768	0.411
	ESM-MSA-1b	0.654	0.441	0.484	0.403	0.866	0.553
	Sequence	0.643	0.450	0.486	0.407	0.865	0.556
	ESMIF	0.601	0.367	0.416	0.315	0.795	0.442
	Structure	0.605	0.375	0.422	0.324	0.804	0.459
	Sequence & structure	0.697	0.454	0.516	0.441	0.880	0.581
RBR_495*	PSSM & HHM	0.501	0.346	0.365	0.259	0.760	0.399
	ESM-MSA-1b	0.623	0.447	0.477	0.397	0.860	0.543
	Sequence	0.603	0.458	0.476	0.399	0.860	0.545
	ESMIF	0.544	0.324	0.367	0.250	0.754	0.390
	Structure	0.550	0.332	0.376	0.265	0.766	0.404
	Sequence & structure	0.677	0.430	0.492	0.410	0.864	0.548
DBR_129	PSSM & HHM	0.590	0.321	0.369	0.344	0.851	0.404
	ESM-MSA-1b	0.748	0.405	0.488	0.481	0.923	0.560
	Sequence	0.735	0.415	0.492	0.485	0.924	0.564
	ESMIF	0.597	0.306	0.379	0.351	0.844	0.426
	Structure	0.557	0.345	0.393	0.366	0.860	0.446
	Sequence & structure	0.727	0.464	0.528	0.521	0.937	0.594
DBR_129*	PSSM & HHM	0.556	0.330	0.364	0.337	0.843	0.391
	ESM-MSA-1b	0.754	0.386	0.476	0.469	0.920	0.546
	Sequence	0.796	0.376	0.472	0.471	0.922	0.554
	ESMIF	0.554	0.268	0.334	0.293	0.801	0.367
	Structure	0.542	0.284	0.344	0.304	0.808	0.383
	Sequence & structure	0.690	0.423	0.491	0.477	0.922	0.552
DBR181	PSSM & HHM	0.570	0.235	0.279	0.268	0.823	0.310
	ESM-MSA-1b	0.674	0.318	0.399	0.399	0.909	0.445
	Sequence	0.663	0.330	0.406	0.404	0.910	0.450
	ESMIF	0.474	0.234	0.286	0.262	0.825	0.315
	Structure	0.489	0.255	0.303	0.284	0.842	0.336
	Sequence & structure	0.661	0.365	0.433	0.431	0.925	0.483

DBR_181*	PSSM & HHM	0.622	0.202	0.266	0.253	0.814	0.283
	ESM-MSA-1b	0.640	0.331	0.401	0.397	0.906	0.434
	Sequence	0.659	0.330	0.406	0.404	0.908	0.437
	ESMIF	0.502	0.192	0.248	0.223	0.779	0.278
	Structure	0.526	0.195	0.256	0.234	0.796	0.289
	Sequence & structure	0.643	0.334	0.409	0.404	0.908	0.448
RBR_117	PSSM & HHM	0.533	0.210	0.258	0.228	0.792	0.295
	ESM-MSA-1b	0.560	0.284	0.332	0.314	0.856	0.387
	Sequence	0.604	0.274	0.336	0.321	0.858	0.389
	ESMIF	0.520	0.210	0.258	0.220	0.768	0.294
	Structure	0.607	0.201	0.263	0.233	0.787	0.313
	Sequence & structure	0.643	0.294	0.365	0.355	0.874	0.419
RBR_117*	PSSM & HHM	0.416	0.232	0.241	0.210	0.778	0.280
	ESM-MSA-1b	0.565	0.275	0.325	0.308	0.850	0.370
	Sequence	0.595	0.271	0.330	0.314	0.853	0.379
	ESMIF	0.468	0.155	0.204	0.152	0.697	0.220
	Structure	0.551	0.153	0.214	0.167	0.718	0.231
	Sequence & structure	0.574	0.271	0.328	0.310	0.851	0.385
RBR_106	PSSM & HHM	0.495	0.260	0.301	0.241	0.766	0.318
	ESM-MSA-1b	0.633	0.338	0.404	0.368	0.853	0.445
	Sequence	0.625	0.345	0.407	0.370	0.855	0.445
	ESMIF	0.573	0.339	0.388	0.341	0.831	0.415
	Structure	0.537	0.363	0.395	0.350	0.840	0.437
	Sequence & structure	0.604	0.425	0.457	0.426	0.877	0.503
RBR_106*	PSSM & HHM	0.475	0.247	0.285	0.224	0.748	0.287
	ESM-MSA-1b	0.639	0.314	0.388	0.349	0.841	0.430
	Sequence	0.666	0.312	0.392	0.357	0.844	0.430
	ESMIF	0.573	0.237	0.305	0.249	0.771	0.328
	Structure	0.408	0.294	0.304	0.255	0.779	0.341
	Sequence & structure	0.542	0.376	0.396	0.361	0.849	0.440

* represents trRosetta-based predicted structures used for evaluation.
