# Supplementary material for: Structure-based prediction of nucleic acid binding residues by merging deep learning- and template-based approaches
Source: PLoS Comput Biol. 2023 Sep 6;19(9):e1011428. doi: 10.1371/journal.pcbi.1011428 (PMC10482303; doi:10.1371/journal.pcbi.1011428)
Supplement: S2 Table — (PDF) [file pcbi.1011428.s010.pdf]

S2 Table. P-values of performance difference between merging module and its two submodules

Dataset	P-values of AUC		P-values of AUPR	
	Mer v.s. DL	Mer v.s. TL	Mer v.s. DL	Mer v.s. TL
DBR_573	1.46e-08	4.29e-13	1.30e-12	5.23e-14
DBR_573*	2.48e-05	2.53e-14	1.92e-07	2.85e-15
DBR_129	2.80e-09	1.14e-11	3.84e-10	1.40e-10
DBR_129*	1.37e-03	4.01e-13	6.72e-04	8.00e-12
DBR_181	9.96e-09	8.99e-12	1.98e-10	5.07e-12
DBR_181*	1.06e-01	6.32e-15	3.09e-06	1.99e-14
RBR_495	5.85e-13	1.57e-04	1.18e-10	2.22e-14
RBR_495*	2.76e-08	1.16e-14	4.16e-09	7.47e-15
RBR_117	7.00e-06	1.57e-04	1.07e-07	9.41e-12
RBR_117*	7.62e-01	1.14e-13	5.24e-02	1.67e-11
RBR_106	9.28e-06	4.87e-14	1.45e-09	1.58e-12
RBR_106*	5.97e-05	3.53e-13	1.21e-04	1.73e-12

* represents trRosetta-based predicted structures used for evaluation.

Mer: merging module, DL: deep learning module, and TL: template module.
