# Supplementary material for: Structure-based prediction of nucleic acid binding residues by merging deep learning- and template-based approaches
Source: PLoS Comput Biol. 2023 Sep 6;19(9):e1011428. doi: 10.1371/journal.pcbi.1011428 (PMC10482303; doi:10.1371/journal.pcbi.1011428)
Supplement: S3 Table — (PDF) [file pcbi.1011428.s011.pdf]

S3 Table. Comparison of NABind and our previous methods on different datasets

Dataset	Method	Recall	Precision	F1	MCC	AUC	AUPR
DBR_573	$DNABind^{ML}$	0.695	0.403	0.442	0.358	0.728	0.461
	$NABind^{DL}$	0.748	0.549	0.606	0.575	0.931	0.695
	$DNABind^{TL}$	0.479	0.466	0.436	0.383	-	-
	$NABind^{TL}$	0.610	0.479	0.502	0.455	0.831	0.561
	DNABind	0.722	0.418	0.463	0.382	0.798	0.532
	$NABind^{Mer}$	0.812	0.538	0.622	0.595	0.939	0.728
	NABind	0.754	0.587	0.634	0.606	0.940	0.729
DBR_573*	$DNABind^{ML}$	0.667	0.390	0.426	0.339	0.718	0.437
	$NABind^{DL}$	0.713	0.532	0.581	0.545	0.918	0.658
	$DNABind^{TL}$	0.412	0.385	0.362	0.293	-	-
	$NABind^{TL}$	0.490	0.405	0.404	0.349	0.774	0.464
	DNABind	0.705	0.376	0.430	0.343	0.769	0.473
	$NABind^{Mer}$	0.759	0.510	0.582	0.549	0.920	0.666
	NABind	0.740	0.524	0.584	0.550	0.920	0.666
RBR_495	$RBRDetector^{ML}$	0.524	0.437	0.428	0.347	0.816	0.448
	$NABind^{DL}$	0.697	0.454	0.516	0.441	0.880	0.581
	$RBRDetector^{TL}$	0.447	0.419	0.391	0.315	-	-
	$NABind^{TL}$	0.539	0.427	0.420	0.347	0.768	0.481
	RBRDetector	0.614	0.446	0.476	0.400	0.839	0.505
	$NABind^{Mer}$	0.723	0.482	0.541	0.473	0.893	0.621
	NABind	0.733	0.474	0.538	0.470	0.894	0.622
RBR_495*	$RBRDetector^{ML}$	0.555	0.385	0.413	0.319	0.792	0.452
	$NABind^{DL}$	0.677	0.430	0.492	0.410	0.864	0.548
	$RBRDetector^{TL}$	0.400	0.368	0.339	0.252	-	-
	$NABind^{TL}$	0.495	0.356	0.352	0.255	0.719	0.405
	RBRDetector	0.611	0.392	0.440	0.349	0.807	0.487
	$NABind^{Mer}$	0.725	0.416	0.493	0.413	0.870	0.561
	NABind	0.662	0.459	0.500	0.424	0.871	0.563

* represents trRosetta-based predicted structures used for evaluation.
