# Supplementary material for: Structure-based prediction of nucleic acid binding residues by merging deep learning- and template-based approaches
Source: PLoS Comput Biol. 2023 Sep 6;19(9):e1011428. doi: 10.1371/journal.pcbi.1011428 (PMC10482303; doi:10.1371/journal.pcbi.1011428)
Supplement: S4 Table — (PDF) [file pcbi.1011428.s012.pdf]

S4 Table. Residue-based performance of NABind and other methods on test sets

Dataset	Method	Recall	Precision	F1	MCC	AUC	AUPR
DBR_129	COACH-D*	0.332	0.299	0.314	0.269	0.670	0.179
	COACH-D	0.367	0.357	0.362	0.321	0.710	0.269
	NCBRPred	0.312	0.392	0.347	0.313	0.823	0.310
	SVMnuc	0.316	0.371	0.341	0.304	0.812	0.302
	NucBind*	0.583	0.248	0.348	0.321	0.822	0.298
	NucBind	0.330	0.381	0.354	0.317	0.811	0.294
	DNABind*	0.709	0.255	0.375	0.367	0.880	0.408
	DNABind	0.680	0.353	0.465	0.447	0.911	0.509
	GraphBind*	0.533	0.377	0.442	0.407	0.882	0.400
	GraphBind	0.684	0.422	0.522	0.500	0.928	0.519
	GraphSite*	0.577	0.465	0.515	0.484	0.924	0.515
	GraphSite	0.606	0.489	0.542	0.512	0.933	0.544
	NABind*	0.719	0.433	0.540	0.522	0.938	0.557
	NABind	**0.754**	**0.525**	**0.619**	**0.601**	**0.961**	**0.672**
DBR_181	COACH-D*	0.239	0.237	0.238	0.204	0.643	0.122
	COACH-D	0.254	0.280	0.266	0.235	0.655	0.172
	NCBRPred	0.414	0.202	0.271	0.235	0.770	0.201
	SVMnuc	0.289	0.242	0.263	0.229	0.803	0.193
	NucBind*	0.537	0.161	0.248	0.237	0.803	0.201
	NucBind	0.293	0.248	0.269	0.234	0.796	0.191
	DNABind*	0.600	0.160	0.252	0.253	0.823	0.210
	DNABind	0.582	0.217	0.317	0.311	0.861	0.293
	GraphBind*	0.376	0.292	0.328	0.297	0.828	0.254
	GraphBind	0.624	0.293	0.399	0.392	0.904	0.339
	GraphSite*	0.458	0.345	0.393	0.366	0.899	0.339
	GraphSite	0.509	0.368	0.427	0.403	0.919	0.380
	NABind*	0.609	0.318	0.417	0.406	0.918	0.408
	NABind	**0.702**	**0.425**	**0.530**	**0.521**	**0.948**	**0.543**
RBR_117	COACH-D*	0.200	0.244	0.220	0.180	0.568	0.114
	COACH-D	0.221	0.252	0.235	0.195	0.663	0.143
	NCBRPred	0.260	0.173	0.208	0.156	0.657	0.124
	NucBind*	0.400	0.170	0.239	0.195	0.725	0.173
	NucBind	0.231	0.235	0.233	0.189	0.715	0.183
	SVMnuc	0.231	0.240	0.235	0.192	0.729	0.184
	aaRNA*	0.404	0.156	0.225	0.182	0.739	0.162
	aaRNA	0.484	0.166	0.247	0.214	0.771	0.170
	NucleicNet*	0.384	0.153	0.218	0.172	0.668	0.124
	NucleicNet	0.371	0.201	0.261	0.216	0.788	0.223
	PSTPRNA*	0.504	0.188	0.274	0.244	0.778	0.188
	PSTPRNA	0.573	0.245	0.343	0.321	0.851	0.262
	RBRDetector*	0.503	0.196	0.282	0.252	0.792	0.218
	RBRDetector	0.269	0.416	0.327	0.304	0.823	0.277

	GraphBind*	0.481	0.191	0.274	0.240	0.761	0.181
	GraphBind	0.463	0.294	0.358	0.322	0.854	0.273
	NABind*	0.545	0.298	0.385	0.357	0.864	0.325
	NABind	**0.682**	**0.316**	**0.432**	**0.421**	**0.905**	**0.424**
RBR_106	COACH-D*	0.128	0.383	0.191	0.182	0.519	0.149
	COACH-D	0.254	0.368	0.301	0.252	0.575	0.196
	NCBRPred	0.199	0.275	0.231	0.176	0.639	0.181
	NucBind*	0.231	0.327	0.271	0.218	0.725	0.249
	NucBind	0.231	0.327	0.271	0.218	0.709	0.246
	SVMnuc	0.394	0.231	0.291	0.213	0.728	0.236
	aaRNA*	0.436	0.215	0.288	0.210	0.729	0.213
	aaRNA	0.451	0.274	0.341	0.271	0.775	0.286
	NucleicNet*	0.328	0.210	0.256	0.173	0.633	0.154
	NucleicNet	0.520	0.329	0.403	0.342	0.747	0.279
	PSTPRNA*	0.339	0.134	0.192	0.088	0.574	0.127
	PSTPRNA	0.646	0.320	0.428	0.381	0.858	0.379
	RBRDetector*	0.472	0.230	0.310	0.237	0.747	0.257
	RBRDetector	0.367	0.382	0.374	0.316	0.804	0.356
	GraphBind*	0.373	0.262	0.307	0.233	0.725	0.236
	GraphBind	0.549	0.373	0.444	0.389	0.835	0.405
	NABind*	0.464	0.361	0.406	0.345	0.835	0.361
	NABind	**0.658**	**0.427**	**0.518**	**0.474**	**0.897**	**0.524**

* represents trRosetta-based predicted protein structures used for evaluation.
