# Supplementary material for: Structure-based prediction of nucleic acid binding residues by merging deep learning- and template-based approaches
Source: PLoS Comput Biol. 2023 Sep 6;19(9):e1011428. doi: 10.1371/journal.pcbi.1011428 (PMC10482303; doi:10.1371/journal.pcbi.1011428)
Supplement: S5 Table — (PDF) [file pcbi.1011428.s013.pdf]

S5 Table. Comparison of NABind and deep learning methods on test sets

Dataset	Method	Recall	Precision	F1	MCC	AUC	AUPR
DBR_129+	NCBRPred	0.590	0.268	0.331	0.300	0.817	0.360
	GraphBind	0.613	0.432	0.457	0.442	0.910	0.533
	GraphSite	0.595	0.462	0.463	0.453	0.921	0.563
	NABind	0.711	0.476	0.529	0.522	0.939	0.614
DBR_181+	NCBRPred	0.441	0.238	0.264	0.240	0.798	0.296
	GraphBind	0.498	0.335	0.357	0.344	0.885	0.391
	GraphSite	0.486	0.355	0.367	0.355	0.906	0.439
	NABind	0.657	0.383	0.446	0.445	0.927	0.506
RBR_117+	NCBRPred	0.262	0.133	0.143	0.118	0.665	0.200
	NucleicNet	0.408	0.205	0.243	0.202	0.693	0.248
	PSTPRNA	0.663	0.172	0.248	0.234	0.789	0.296
	GraphBind	0.615	0.204	0.264	0.248	0.823	0.309
	NABind	0.608	0.278	0.341	0.329	0.866	0.411
RBR_106+	NCBRPred	0.233	0.224	0.171	0.145	0.673	0.255
	NucleicNet	0.394	0.274	0.299	0.244	0.691	0.286
	PSTPRNA	0.606	0.268	0.354	0.320	0.805	0.368
	GraphBind	0.435	0.217	0.256	0.205	0.722	0.298
	NABind	0.592	0.406	0.446	0.412	0.877	0.490

+ represents AlphaFold2-based predicted protein structures used for evaluation.
