# Supplementary material for: Structure-based prediction of nucleic acid binding residues by merging deep learning- and template-based approaches
Source: PLoS Comput Biol. 2023 Sep 6;19(9):e1011428. doi: 10.1371/journal.pcbi.1011428 (PMC10482303; doi:10.1371/journal.pcbi.1011428)
Supplement: S6 Table — (PDF) [file pcbi.1011428.s014.pdf]

S6 Table. Residue-based performance of NABind and deep learning methods on test sets

Dataset	Method	Recall	Precision	F1	MCC	AUC	AUPR
DBR_129^{+}	NCBRPred	0.312	0.392	0.347	0.313	0.823	0.310
	GraphBind	0.625	0.434	0.512	0.484	0.916	0.497
	GraphSite	0.665	0.460	0.543	0.519	0.934	0.544
	NABind	0.756	0.483	0.589	0.573	0.953	0.633
DBR_181^{+}	NCBRPred	0.414	0.202	0.271	0.235	0.770	0.201
	GraphBind	0.505	0.304	0.380	0.357	0.893	0.317
	GraphSite	0.517	0.354	0.420	0.397	0.917	0.369
	NABind	0.684	0.372	0.482	0.476	0.938	0.487
RBR_117^{+}	NCBRPred	0.260	0.173	0.208	0.156	0.657	0.124
	NucleicNet	0.442	0.195	0.270	0.234	0.725	0.172
	PSTPRNA	0.695	0.174	0.279	0.280	0.809	0.225
	GraphBind	0.547	0.172	0.262	0.239	0.787	0.188
	NABind	0.621	0.301	0.406	0.388	0.887	0.369
RBR_106^{+}	NCBRPred	0.199	0.275	0.231	0.176	0.639	0.181
	NucleicNet	0.401	0.259	0.315	0.254	0.710	0.203
	PSTPRNA	0.636	0.256	0.365	0.330	0.814	0.309
	GraphBind	0.421	0.220	0.289	0.226	0.722	0.223
	NABind	0.605	0.389	0.473	0.434	0.893	0.438

$^{+}$ represents AlphaFold2-based predicted protein structures used for evaluation.
