# Supplementary material for: Structure-based prediction of nucleic acid binding residues by merging deep learning- and template-based approaches
Source: PLoS Comput Biol. 2023 Sep 6;19(9):e1011428. doi: 10.1371/journal.pcbi.1011428 (PMC10482303; doi:10.1371/journal.pcbi.1011428)
Supplement: S7 Table — (PDF) [file pcbi.1011428.s015.pdf]

S7 Table. Performance of different modules on test sets

Dataset	Classifier	Recall	Precision	F1	MCC	AUC	AUPR
DBR_129	$NABind^{DL}$	0.727	0.464	0.528	0.521	0.937	0.594
	$NABind^{TL}$	0.581	0.367	0.417	0.392	0.804	0.475
	$NABind^{Mer}$	0.777	0.445	0.536	0.532	0.945	0.637
	NABind	0.700	0.504	0.541	0.535	0.946	0.637
DBR_129*	$NABind^{DL}$	0.690	0.423	0.491	0.477	0.922	0.552
	$NABind^{TL}$	0.468	0.297	0.331	0.292	0.765	0.380
	$NABind^{Mer}$	0.725	0.404	0.488	0.478	0.925	0.557
	NABind	0.708	0.420	0.494	0.482	0.925	0.560
DBR_181	$NABind^{DL}$	0.661	0.365	0.433	0.431	0.925	0.483
	$NABind^{TL}$	0.538	0.297	0.357	0.339	0.803	0.408
	$NABind^{Mer}$	0.748	0.374	0.466	0.472	0.936	0.548
	NABind	0.658	0.432	0.484	0.481	0.937	0.550
DBR_181*	$NABind^{DL}$	0.643	0.334	0.409	0.404	0.908	0.448
	$NABind^{TL}$	0.363	0.220	0.245	0.218	0.714	0.293
	$NABind^{Mer}$	0.665	0.323	0.404	0.403	0.909	0.463
	NABind	0.632	0.330	0.405	0.400	0.909	0.464
RBR_117	$NABind^{DL}$	0.643	0.294	0.365	0.355	0.874	0.419
	$NABind^{TL}$	0.427	0.202	0.239	0.200	0.699	0.304
	$NABind^{Mer}$	0.644	0.311	0.378	0.368	0.881	0.455
	NABind	0.659	0.306	0.377	0.368	0.882	0.457
RBR_117*	$NABind^{DL}$	0.574	0.271	0.328	0.310	0.851	0.385
	$NABind^{TL}$	0.291	0.151	0.172	0.118	0.627	0.201
	$NABind^{Mer}$	0.610	0.252	0.324	0.306	0.851	0.381
	NABind	0.546	0.280	0.330	0.311	0.852	0.383
RBR_106	$NABind^{DL}$	0.604	0.425	0.457	0.426	0.877	0.503
	$NABind^{TL}$	0.409	0.312	0.312	0.256	0.733	0.368
	$NABind^{Mer}$	0.621	0.447	0.476	0.446	0.883	0.541
	NABind	0.638	0.443	0.479	0.449	0.884	0.542
RBR_106*	$NABind^{DL}$	0.542	0.376	0.396	0.361	0.849	0.440
	$NABind^{TL}$	0.326	0.240	0.229	0.176	0.687	0.280
	$NABind^{Mer}$	0.588	0.366	0.404	0.369	0.854	0.449
	NABind	0.507	0.391	0.393	0.357	0.854	0.452

* represents trRosetta-based predicted protein structures used for evaluation.
