# Supplementary material for: Structure-based prediction of nucleic acid binding residues by merging deep learning- and template-based approaches
Source: PLoS Comput Biol. 2023 Sep 6;19(9):e1011428. doi: 10.1371/journal.pcbi.1011428 (PMC10482303; doi:10.1371/journal.pcbi.1011428)
Supplement: S8 Table — (PDF) [file pcbi.1011428.s016.pdf]

S8 Table. Performance of cross-prediction for DNA- and RNA-binding residues

Dataset	Recall	Precision	F1	MCC	AUC	AUPR
DBR_129	0.775	0.414	0.509	0.506	0.940	0.599
DBR_181	0.756	0.342	0.437	0.447	0.933	0.537
RBR_117	0.522	0.377	0.383	0.369	0.879	0.446
