# Supplementary material for: Structure-based prediction of nucleic acid binding residues by merging deep learning- and template-based approaches
Source: PLoS Comput Biol. 2023 Sep 6;19(9):e1011428. doi: 10.1371/journal.pcbi.1011428 (PMC10482303; doi:10.1371/journal.pcbi.1011428)
Supplement: S9 Table — (PDF) [file pcbi.1011428.s017.pdf]

S9 Table. A summary of datasets used in this study

Dataset	Number of proteins	Number of binding residues	Number of non-binding residues	Proportion of binding residues
DBR_573	573	14479	145404	9.06%
DBR_129	129	2240	35275	5.97%
DBR_181	181	3208	72050	4.26%
RBR_495	495	14609	122290	10.67%
RBR_117	117	2031	35314	5.44%
RBR_106	106	3422	35843	8.72%
