# Supplementary material for: Structure-based prediction of nucleic acid binding residues by merging deep learning- and template-based approaches
Source: PLoS Comput Biol. 2023 Sep 6;19(9):e1011428. doi: 10.1371/journal.pcbi.1011428 (PMC10482303; doi:10.1371/journal.pcbi.1011428)
Supplement: S10 Table — (PDF) [file pcbi.1011428.s018.pdf]

S10 Table. Sequence identity between structures from AlphaFold database and native structures

Dataset	PDB ID	UniProt accession	Sequence length	Identity (%)
RBR_117	3jcm_B	P20053	429	100
	3jcm_G	P19735	734	100
	3jcm_I	P49704	416	100
	4xbf_A	O60341	666	100
	4z7l_A	A4FXZ3	218	100
	5axm_B	A0A1C7D1G9	239	100
	5d0a_A	P97030	379	100
	5do4_H	P00734	256	100
	5ed1_A	P78563	396	99.7
	5g2x_C	P0A3U0	486	100
	5gan_D	Q06819	140	100
	5hr7_B	P36979	358	99.7
	5jb3_8	Q97W59	129	100
	5k36_I	P53859	224	100
	5kal_A	Q381M1	325	100
	5kl1_B	P25724	70	100
	5lj3_D	P28320	114	100
	5lj3_G	P21374	97	100
	5lj3_T	Q04048	592	99.8
	5lm7_B	P0A780	134	100
	5lmn_X	Q5SKU2	168	100
	5mps_R	Q03375	108	100
	5n94_A	Q8SWT2	849	100
	5o1y_A	P53617	163	100
	5o7h_D	A4Y6G1	310	100
	5o7h_F	A4Y6G2	326	100
	5o9z_E	O43395	219	100
	5o9z_G	O94906	804	100
	5o9z_N	Q96NC0	56	100
	5oa3_0	P41214	479	100
	5osg_h	A0A3Q8IT45	173	100
	5ud5_A	Q8PWY1	86	100
	5uz9_A	Q02ML9	424	100
	5uz9_B	Q02MM0	305	100
	5uz9_F	Q02MM1	335	100
	5v7c_B	Q6PKG0	144	100
	5vt0_L	P00579	471	100
	5wqe_A	T0D7A2	992	99.9
	5wt1_A	Q9V2G1	332	100
	5wwr_A	Q8TEA1	461	100
	5wzg_A	Q9C552	534	100
	5x6b_F	Q58876	177	100

5xj2_A	Q97R12	454	99.8
5y58_A	P32807	548	100
5y58_B	Q04437	568	100
5z3g_M	P53261	371	100
5z3g_P	Q12690	108	100
5z9x_A	A3KPE8	288	100
5zw4_A	O32036	216	100
5zwn_Y	Q07508	196	100
6ah3_B	P41812	784	100
6ah3_D	P38336	203	100
6ah3_E	P28005	146	100
6ah3_J	P38786	293	100
6ah3_K	P40571	128	100
6ahu_B	Q99575	774	100
6ahu_J	P78346	247	100
6ahu_K	Q9H633	121	100
6c0f_7	P40007	156	100
6c0f_K	P38779	260	100
6c0f_o	P53927	133	100
6d12_B	Q4G0J3	101	98
6dnh_B	Q9C0J8	376	100
6du4_A	Q86W50	287	100
6dzp_g	A0R554	64	100
6e9f_A	B0MS50	864	99.5
6exn_H	P53333	411	100
6exn_N	P38241	242	100
6exn_O	Q03654	322	100
6exn_P	Q03772	73	100
6exn_S	Q12309	472	100
6exn_a	P33411	171	100
6exn_y	P53277	88	100
6f3h_B	Q6FJE0	777	99.9
6f4g_D	Q9V4Q8	176	98.3
6ff4_7	Q15428	93	100
6ff4_E	O60508	130	100
6ff4_P	Q9NW64	286	100
6ff4_t	O15541	172	100
6ff4_u	O75533	881	100
6fpx_A	O74958	177	100
6fq3_A	E7FAM5	390	100
6g90_Q	Q02554	220	100
6g90_S	Q06835	103	100
6g90_T	P19736	462	100
6g90_W	Q08963	170	100

RBR_106	5oc6_A	Q9NX74	90	100
	5zkj_B	Q9Y2C4	303	99.7
	6dtd_A	E6K398	1053	100
	6e0o_A	A0A4V8GZR7	273	100
	6ij2_A	A0A0H3AJ04	236	100
	6koo_F	P9WGH9	182	100
	6l1w_A	Q3UPF5	215	100
	6lvr_C	Q66GI4	194	97.9
	6m6r_A	Q93413	166	100
	6m6v_A	Q8ECH7	117	100
	6m6v_B	Q8ECH6	132	100
	6m7k_A	Q8VC28	318	99.1
	6ozj_B	Q8C9A2	250	100
	6ppq_E	O42978	75	100
	6r7b_A	F0NE21	454	100
	6rti_A	Q04609	693	100
	6s0m_B	A0A045JQ63	158	100
	6snj_A	P35637	131	100
	6sty_A	Q9Y3B8	198	100
	6sy4_A	P04483	188	94.1
	6tyg_F	P9WGH5	147	100
	6u6y_B	Q9Y3Z3	437	99.8
	6u9x_A	B6SBM0	294	100
	6uso_A	Q0QHL8	596	100
	6vm6_B	C0VHC9	440	100
	6w11_A	Q97Y88	212	100
	6wlh_A	P19544	118	100
	6wyb_A	D0VWU9	739	97.7
	6xl1_A	F2NWD3	373	99.7
	6y4b_A	G2JBB2	255	100
	6ywo_A	G2R014	354	100
	6yym_A	P08965	148	100
	6zdu_A	A0A1D8PEA0	693	100
	6zww_C	P43329	749	100
	7a9w_A	P53140	513	100
	7c06_A	Q09176	193	100
	7c45_A	Q38DE2	295	100
	7dic_A	P47350	646	99.8
	7e8o_A	A0A1G2XP69	194	100
	7f36_A	Q8ZL98	159	99.4
	7jtq_A	P00751	703	100
	7k9d_A	Q9KGD7	89	100
	7mjy_A	A0A174NUT3	448	100
	7mpl_A	X5MEI1	206	100

7mw8_C	A0A7X9YQB3	385	99.5
7ndj_B	Q5DTV4	192	99.5
7nq4_B	Q9H974	381	100
7og0_B	Q4QL73	498	100
7oyr_A	P00918	257	100
7p8q_A	P37634	275	99.3
7pdv_E	P52756	107	98.1
7pvk_A	Q7MVV4	513	99.8
7qde_B	Q01560	169	100
7r97_A	P0A7Y0	226	98.7
7r9g_A	Q12211	386	99.7
7uj1_B	P23246	270	100
7vti_A	A0A660UUL5	736	99.5
7wkv_A	Q6P6C2	219	100
7wnu_B	P9WGZ9	554	100
7yse_B	P00960	299	100
7yse_D	P00961	643	100
7zgv_A	A0A2I5TBB8	246	100
7zhh_A	Q9VSK3	219	100
8af0_A	P03950	123	99.2
