# Supplementary material for: Structure-based prediction of nucleic acid binding residues by merging deep learning- and template-based approaches
Source: PLoS Comput Biol. 2023 Sep 6;19(9):e1011428. doi: 10.1371/journal.pcbi.1011428 (PMC10482303; doi:10.1371/journal.pcbi.1011428)
Supplement: S11 Table — (PDF) [file pcbi.1011428.s019.pdf]

S11 Table. Alignment scores of secondary structures

Secondary structure	Coil	α-Helix	β-Strand
Coil	1.0	0.0	0.5
α-Helix	0.0	2.0	-0.5
β-Strand	0.5	-0.5	2.0
