# Supplementary material for: Structure-based prediction of nucleic acid binding residues by merging deep learning- and template-based approaches
Source: PLoS Comput Biol. 2023 Sep 6;19(9):e1011428. doi: 10.1371/journal.pcbi.1011428 (PMC10482303; doi:10.1371/journal.pcbi.1011428)
Supplement: S12 Table — (PDF) [file pcbi.1011428.s020.pdf]

S12 Table. Hyper-parameters for different modules

Module	Hyper-parameter	Value
Deep-learning module	Epoch	50
	Learning rate	1e-4
	Loss function	Cross-entropy
	Weight of positive examples	50
	Gradient descent	Nesterov accelerated gradient
	Dropout	0.5
Template module	n_estimators for random forest	500
	n_estimators for XGBoost	100
	n_estimators for LGBM	100
Merging module	n_estimators for LGBM	100
